# Clinical-transcriptomic classification of lumbar disc degeneration enhanced by machine learning

**DOI:** 10.1186/s40779-025-00637-9

**Published:** 2025-08-29

**Authors:** Huai-Jian Jin, Peng Lin, Xiao-Yuan Ma, Sha Huang, Liang Zhang, Ou Hu, Yang-Yang Li, Ying-Bo Wang, Jun Zhu, Bo Hu, Jun-Gang Pu, Qin Qin, Pu-Lin Yan, Bing Liu, Yu Lan, Lin Chen, Yang-Li Xie, Jian He, Yi-Bo Gan, Peng Liu

**Affiliations:** 1 Department of Spine Surgery, Center of Orthopedics, State Key Laboratory of Trauma and Chemical Poisoning, Army Medical Center of PLA (Daping Hospital), Army Medical University, Chongqing, 400042 China; 2 Department of Wound Infection and Drug, State Key Laboratory of Trauma and Chemical Poisoning, Army Medical Center of PLA (Daping Hospital), Army Medical University, Chongqing, 400042 China; 3 Pain Department, Army Medical Center of PLA (Daping Hospital), Army Medical University, Chongqing, 400042 China; 4 State Key Laboratory of Experimental Hematology, Haihe Laboratory of Cell Ecosystem, Senior Department of Hematology, Fifth Medical Center, Medical Innovation Research Department, Chinese PLA General Hospital, Beijing, 100071 China; 5 State Key Laboratory of Experimental Hematology, Haihe Laboratory of Cell Ecosystem, Key Laboratory for Regenerative Medicine of Ministry of Education, Institute of Hematology, School of Medicine, Jinan University, Guangzhou, 510632 China; 6 Center of Bone Metabolism and Repair, State Key Laboratory of Trauma and Chemical Poisoning, Trauma Center, Research Institute of Surgery, Laboratory for the Prevention and Rehabilitation of Military Training Related Injuries, Army Medical Center of PLA (Daping Hospital), Army Medical University, Chongqing, 400042 China; 7 Tissue Stress Injury and Functional Repair Key Laboratory of Sichuan Province, The General Hospital of Western Theater Command, Chengdu, 610031 China

**Keywords:** Lumbar disc degeneration (LDD), Molecular classification, Machine learning, Diagnosis, Transcriptome, RNA sequencing (RNA-seq)

## Abstract

**Background:**

Lumbar disc degeneration (LDD) displays considerable heterogeneity in terms of clinical features and pathological changes. However, researchers have not clearly determined whether the transcriptome variations in LDD could be used to identify or interpret the causes of heterogeneity in clinical features. This study aimed to identify the transcriptomic classification of degenerated discs in LDD patients and whether the molecular subtypes of LDD could be accurately predicted using clinical features.

**Methods:**

One hundred and twenty-two nucleus pulposus (NP) tissues from 108 patients were consecutively collected for bulk RNA sequencing (RNA-seq). An unsupervised clustering method was employed to analyze the bulk RNA matrix. Differential analysis was performed to characterize the transcriptional signatures and subtype-specific extracellular matrix (ECM) dysregulation. The cell subpopulation states of each subtype were inferred by integrating bulk and single-cell sequencing datasets. Transwell and dual-luciferase reporter gene assays were employed to investigate possible molecular mechanisms involved. Machine learning algorithm diagnostic prediction models were developed to correlate molecular classification with clinical features.

**Results:**

LDD was classified into 4 subtypes with distinct molecular signatures and ECM remodeling: C1 with collagenesis, C2 with ossification, C3 with low chondrogenesis, and C4 with fibrogenesis. Chond1-3 in C1 dominated disc collagenesis via the activation of the mechanosensors *TRPV4* and *PIEZO1*; NP progenitor cells in C2 exhibited chondrogenic and osteogenic phenotypes; Chond1 in C3 was linked to a disrupted hypoxic microenvironment leading to reduced chondrogenesis; Macrophages in C4 played a crucial role in disc fibrogenesis via the secretion of tumor necrosis factor-α (TNF-α). Furthermore, the random forest diagnostic prediction model was proven to have a robust performance [area under the receiver operating characteristic (ROC) curve: 0.9312; accuracy: 0.84] in stratifying the molecular subtypes of LDD based on 12 clinical features.

**Conclusions:**

Our study delineates 4 distinct molecular subtypes of LDD that can be accurately stratified on the basis of clinical features. The identification of these subtypes would facilitate precise diagnostics and guide the development of personalized treatment strategies for LDD.

**Supplementary Information:**

The online version contains supplementary material available at 10.1186/s40779-025-00637-9.

## Background

Low back pain is a prevalent and complex condition of the musculoskeletal system that primarily results from lumbar disc degeneration (LDD). The global prevalence of LDD is estimated to reach as high as 60% [1], significantly impairing patients’ physical and mental health, while also imposing heavy socioeconomic burdens [2]. Understanding the underlying molecular mechanisms of disc degeneration is crucial, as it directly impacts patient prognosis and treatment options, increasing the potential for personalized therapeutic approaches. Traditional treatments focus primarily on pain alleviation without addressing the underlying pathophysiology, which is due to the insufficient understanding of the pathological mechanisms involved in patient stratification [3]. This gap hinders the translation of promising biotherapeutic approaches from the bench to the bedside, particularly regarding personalized strategies for effective treatment.

Currently, LDD is classified on the basis of radiological and histological characteristics, including the Schneiderman score [4], Thompson grade [5], Boos classification [6], and Pfirrmann grade [7]. While these classifications can describe the severity of disc degeneration, they fail to accurately diagnose the heterogeneous pathologies of LDD or guide personalized treatment [8, 9]. Emerging evidence has highlighted the significant heritability of LDD, which also exhibits pleiotropy with risk factors for other diseases such as osteoporosis [10, 11]. Nevertheless, the potential of molecular signatures derived from gene expression data for enhancing LDD diagnosis, prognosis, or therapeutic interventions remains to be elucidated. Recent advances in molecular classification, particularly those based on transcriptome analysis, have shown potential for more precise diagnostic and treatment approaches in disorders such as osteoarthritis and rheumatoid arthritis [12, 13]. Although several efforts have been made to elucidate the molecular heterogeneity of different Thompson or Pfirrmann grades of LDD [14], there is an urgent need to establish transcriptomic molecular classification of LDD to facilitate diagnostic stratification, which would enhance the implementation of tailored biotherapeutic interventions.

In this study, we aimed to bridge the gap from the molecular heterogeneity of LDD to patient stratification for biotherapeutic interventions. We developed a comprehensive molecular classification of LDD on the basis of transcriptome analysis, revealing biomarkers and pathological phenotypes across distinct subtypes. We further explored the relationships between these subtypes and clinical features, utilizing machine learning to develop diagnostic prediction models for clinical applications. Additionally, we predicted and validated the cellular composition of each subtype through integrated analysis of bulk RNA sequencing (RNA-seq) and single-cell transcriptomic datasets of LDD. Through this approach, we illuminated the possible pathological mechanisms underlying inflammatory damage to nucleus pulposus cells (NPCs) in the C4 subtype. These findings could contribute to a deeper understanding of the heterogeneous pathological mechanisms involved in LDD and promote personalized therapeutic strategies.

## Materials and methods

### Study design and patient enrollment

**Fig. 1:**
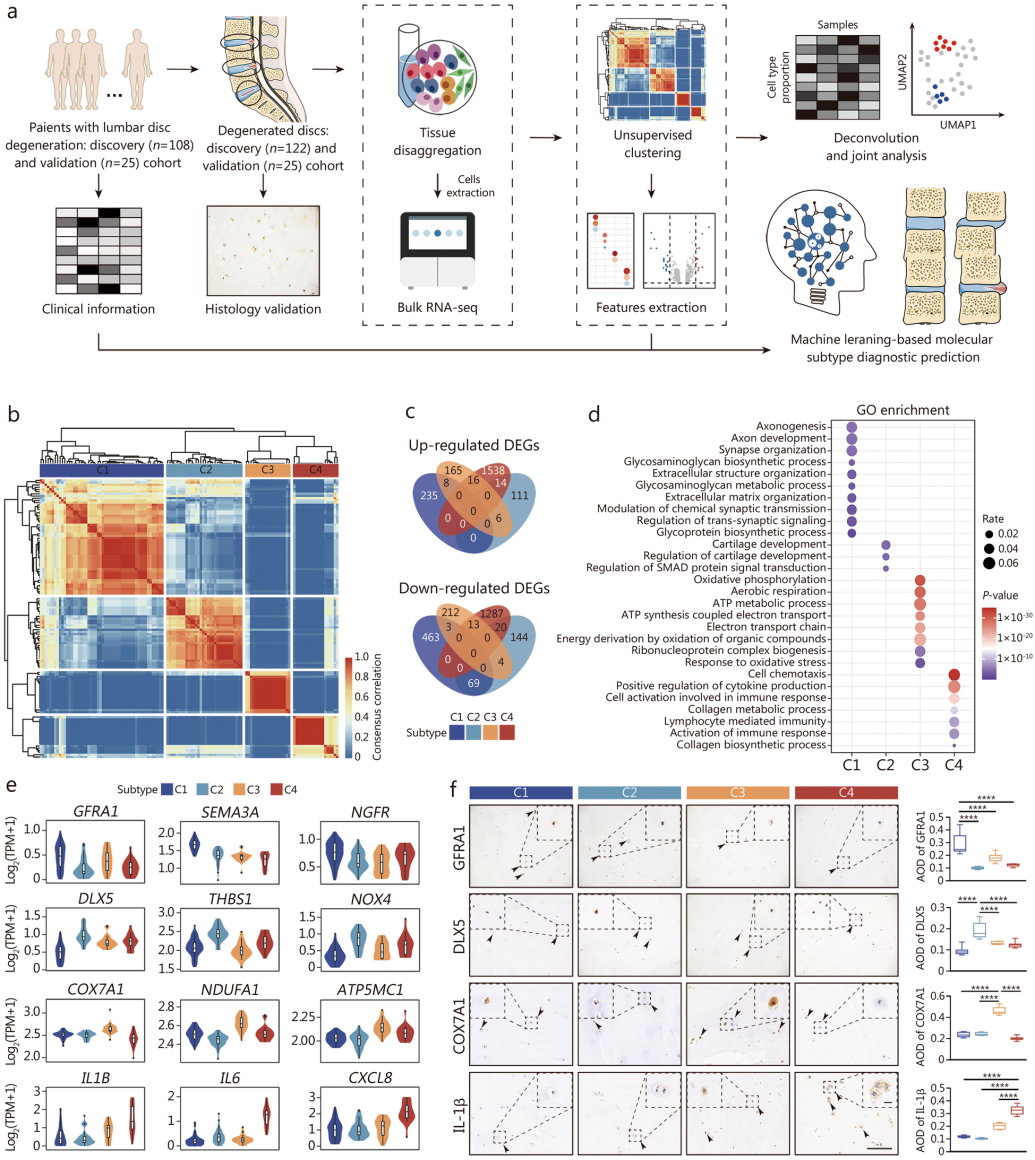
Identification and validation of transcriptome-based LDD subtypes. **a** Study scheme overview. Description of patient recruitment, IVD sample collection, RNA extraction and sequencing, and the computational analysis strategy. **b** Heatmap visualization of genes across 122 NP samples revealing four distinct subtypes (C1–C4). **c** Venn diagram showing upregulated DEGs per subtype. **d** GO analysis showing the enriched biological processes of each subtype. **e** Violin plots showing the expression of representative genes across subtypes. **f** Representative immunohistochemical analysis of the specific selected proteins in NP tissues by subtype. Scale bar = 100 μm, insert panel = 10 μm. ^****^P < 0.0001. AOD average optical density, C1 cluster 1, C2 cluster 2, C3 cluster 3, C4 cluster 4, DEGs differentially expressed genes, GO Gene Ontology, IVD intervertebral disc degeneration, LDD lumbar disc degeneration, NP nucleus pulposus

Multiple steps were designed to develop a comprehensive molecular classification system for LDD (Fig. 1a). We prospectively enrolled patients and collected intervertebral disc (IVD) samples to establish both discovery and validation cohorts. The complete method is provided in Additional file 1.

LDD was confirmed via lumbar magnetic resonance imaging (MRI). Enrolled patients had low back pain and/or sciatica and had no prior surgical intervention affecting the responsible IVD, spinal tumors, spinal infection, or concomitant conditions rendering them not suitable for this study. Demographic and epidemiological data and clinical characteristics were collected. IVD specimens were collected from patients undergoing discectomy and/or intervertebral fusion at Army Medical Center of PLA to establish 3 distinct cohorts: i) a discovery cohort for molecular clustering and development of machine learning-based diagnostic prediction models; ii) a validation cohort for independent verification of the seleceted model; and iii) an individual cohort for flow cytometry analysis. Nucleus pulposus (NP) tissues were identified by microscopy, and sufficient NP tissue to meet the minimum requirements for bulk RNA-seq was collected. NPC isolation, RNA extraction, and RNA-seq library preparation of the NP samples were performed according to the protocols in Additional file 1: Materials and methods. Informed consent was obtained from all patients.

### Disc morphology definition and imaging scores

An MRI scan of the lumbar spine with T1-weighted and T2-weighted sequences was performed using a 1.5-Tesla system (Signa EXCITE; General Electric Health Care, United States). The disc morphology was subclassified into 4 groups by combining lumbar MRI data and intraoperative validation. Briefly, according to the lumbar disc nomenclature recommended by the North American Spine Society, the American Society of Spine Radiology, and the American Society of Neuroradiology, the disc morphology was subclassified as no disc herniation (normal), bulge, contained herniation, or uncontained herniation [15]. The Pfirrmann grade of disc degeneration, Modic changes, and Schmorl’s nodes were assessed. Detailed information is listed in Additional file 2: Table S1.

### Unsupervised clustering

Transcripts per million were used to normalize the expression of each gene. To minimize noise effects, genes with a count greater than 5 in at least 10% of the NP samples were defined as contributing genes [12, 16] and were kept for the following analysis. The normalized count matrix was then used to cluster the NP samples using the unsupervised single-cell consensus clustering (SC3) method [17], which combines multiple clustering solutions in the R/Bioconductor package “SC3”. Contributing genes with the top *n* highest standard deviations were considered highly variable genes (HVGs) which exhibits biological variation beyond technical noise [12]. To determine the appropriate number of HVGs, we tested top *n* HVGs (*n* ranging from 2500 to 11,000 in 500-interval increments) duing downstream clustering analysis. To select the appropriate clustering parameter *k* in SC3, *k* values from 2 to 8 were tested iteratively. For each SC3 run with different *k* values and the top *n* HVGs, the silhouette coefficient was calculated, the consensus matrix was plotted, and cluster-specific genes were identified (Additional file 1: Fig. S1a-c). Although *k* = 2 was associated with a better silhouette coefficient across all groups, it displayed lower clinical relevance and might not comprehensively reveal the underlying molecular classification. The best silhouette coefficient was observed when *n* = 8000 and *k* = 4 (Additional file 1: Fig. S1a); thus, *n* = 8000 and *k* = 4 were identified as the best parameters. The detailed clustering results are listed in Additional file 3.

### Machine learning model development and interpretation

Least absolute shrinkage and selection operator (LASSO) regression analysis was conducted to select 12 clinical features (including dummy variables) from all 23 independent variables. The samples were divided into a training set (*n* = 85) and a testing set (*n* = 37) at a 7 : 3 ratio. Random forest (RF), support vector machine (SVM), eXtreme gradient boosting (XGBoost), multinomial logistic regression (MLR), and neural network (NNet) classification models were built. Tenfold cross-validation was performed to choose the best parameter of each machine learning model. Receiver operating characteristic (ROC) and precision-recall (PR) curves were plotted. The area under the ROC curve (AUROC) and area under the PR curve (AP) were calculated to evaluate model performance. The model with the highest values was selected as optimal. Next, we performed decision curve analysis (DCA) in the R function (rmda 1.6) to evaluate the clinical benefits of the selected model and measure the net benefits of different threshold probabilities in the 122-sample cohort. The Shapley additive explanation (SHAP) interpretation model was built to interpret the whole cohort and a single sample.

### Statistical analysis

Data were analyzed by SPSS 22.0 (IBM), GraphPad Prism 9 (v9.5.1(733)), and R Studio (v4.1.3). The Kolmogorov-Smirnov test was used to assess the normality of data distributions. Student’s *t*-test, ANOVA, and the Wilcoxon test were used to analyze continuous variables. Fisher’s exact test and the chi-square test were used to analyze categorical variables. The data in bar graphs are presented as the mean ± standard deviation (SD) as indicated in the figure legends. Each immunostaining experiment was repeated at least three times with biologically independent samples. *P* < 0.05 was considered to indicate a statistically significant difference. For semiquantitative examination of marker expression, immunostaining results were analyzed using ImageJ software. For all images, the diaminobenzidine or fluorescence intensity within or around the cell nuclei was analyzed. Stained tissue samples were quantified in at least 5 random fields per section and 3 sections per group.

## Results

### Clinical characteristics of the discovery cohort

The discovery cohort comprised 122 NP tissue samples from 108 patients. In all, 94 patients each contributed a single NP sample, whereas the remaining 14 patients each provided 2 distinct samples from different levels, for a total of 28 samples (Additional file 2: Tables S1, S2). The clinical profiles of this cohort are comprehensively described in Table 1. Briefly, the mean age of this cohort was 59.2 years. Among these samples, 59.8% were from male patients, and 77.8% were collected from patients over 50 years of age. The Pfirrmann grade distribution was as follows: 1.6% grade II, 21.3% grade III, 68.9% grade IV, and 8.2% grade V. Regarding disc morphology, 15.6% of samples were classified as normal, 41.8% as bulge, 26.2% as contained herniation, and 16.4% as uncontained herniation. Additionally, 90.1% of samples were located at the L4-5 and L5-S1 levels.

**Table 1:** Characteristics of 122 NP samples in the prospective discovery cohort, including 108 LDD patients

Characteristics	Value
Sex [*n* (%)]	
Male	73 (59.8)
Female	49 (40.2)
Age (year, mean ± SD)	59.2 ± 13.4
Age group	
< 30	5 (4.1)
30–40	6 (4.9)
40–50	16 (13.1)
50–60	33 (27.1)
60–70	31 (25.4)
≥ 70	31 (25.4)
Height (cm, mean ± SD)	163.8 ± 9.2
Weight (kg, mean ± SD)	66.1 ± 11.6
BMI (kg/m^2^, mean ± SD)	24.6 ± 3.5
Hypertension [*n* (%)]	
Yes	36 (29.5)
No	86 (70.5)
Diabetes mellitus [*n* (%)]	
Yes	13 (10.7)
No	109 (89.3)
Smoking [*n* (%)]	
Yes	19 (15.6)
No	103 (84.4)
Drinking (alcohol use) [*n* (%)]	
Yes	16 (13.1)
No	106 (86.9)
Course of disease (month, median)	12
NRS of lower back pain (mean ± SD)	5.6 ± 2.0
NRS of sciatica (mean ± SD)	6.5 ± 1.6
ODI (%, mean ± SD)	45.7 ± 16.3
Numbness [*n* (%)]	
Yes	65 (53.3)
No	57 (46.7)
Neurogenic claudication [*n* (%)]	
Yes	43 (35.2)
No	79 (64.8)
SLR test [*n* (%)]	
Yes	70 (57.4)
No	52 (42.6)
Radiographic parameters	
Spondylolisthesis [*n* (%)]	
Yes	27 (22.1)
No	95 (77.9)
Osteophyte [*n* (%)]	
Yes	90 (73.8)
No	32 (26.2)
IDH (mm, mean ± SD)	10.9 ± 2.7
Lumbar lordosis (°, mean ± SD)	36.4 ± 14.3
Segmental lumbar lordosis (°, mean ± SD)	6.5 ± 4.7
Pfirrmann grade [*n* (%)]	
II	2 (1.6)
III	26 (21.3)
IV	84 (68.9)
V	10 (8.2)
Disc morphology [*n* (%)]	
Normal	19 (15.6)
Bulge	51 (41.8)
Contained herniation	32 (26.2)
Uncontained herniation	20 (16.4)
Modic change [*n* (%)]	
Normal	97 (79.5)
I	4 (3.3)
II	21 (17.2)
Schmorl’s node [*n* (%)]	
Yes	10 (8.2)
No	112 (91.8)
Anatomical level [*n* (%)]	
L4–5	72 (59)
L5–S1	38 (31.1)
L3–4	10 (8.3)
L1–2	1 (0.8)
T12–L1	1 (0.8)

*NP* nucleus pulposus, *LDD* lumbar disc degeneration, *SD* standard deviation, *BMI* body mass index, *NRS* numerical rating scale, *ODI* Oswestry disability index, *SLR* straight-leg-raising, *IDH* intervertebral disc height

### Transcriptome-based molecular classification of 4 distinct LDD subtypes

To identify potential molecular subtypes of degenerative discs from a high-dimensional matrix, SC3 [17] and other clustering solutions were employed. Based on the top 8000 HVGs, 122 NP samples were classified into 4 robust subtypes: 52 (42.6%) into cluster 1 (C1), 32 (26.2%) into cluster 2 (C2), 19 (15.6%) into cluster 3 (C3), and 19 (15.6%) into cluster 4 (C4) (Fig. 1b; Additional file 1: Fig. S1a, b; Additional file 2: Table S1). Surprisingly, of the 28 samples from the 14 patients, both samples from a single patient at different spinal levels were clustered into the same molecular subtype for 16 samples from 8 patients; for the remaining 12 samples from 6 patients, both samples from a single patient exhibited 2 divergent molecular subtypes (Additional file 2: Table S2). These results suggest that discs from different levels of the same LDD patient can present subtype heterogeneity.

To investigate the biological processes of the 4 LDD subtypes, limma [18] was used to identify differentially expressed genes (DEGs) among C1, C2, C3, and C4. Overall, C1, C2, C3, and C4 exhibited 235, 111, 165, and 1,538 uniquely upregulated DEGs and 463, 144, 212, and 1,287 downregulated DEGs, respectively, with cutoffs of log_2_ fold change greater than 0.5 and *P* < 0.05 (Fig. 1c). Gene ontology (GO) analysis revealed that the biological processes of the identified clusters were as follows: C1 was related to axon development and glycosaminoglycan metabolism; C2 was related to chondrocyte development; C3 was related to aerobic metabolism and energy consumption; and C4 was related to inflammation, the immune response and collagen metabolism (Fig. 1d). Subtype-specific upregulated DEGs were visualized via a heatmap (Additional file 1: Fig. S1c). Notably, neuropathic pain mediators (*NPY1R* and *NPY5R*) [19] and factors involved in axonal growth (*SEMA3A*, *NGF*, and *GFRA1*) [20, 21] were enriched in C1, whereas signaling molecules involved in bone and cartilage development (*DLX5*, *BMP2*, *SMAD2*, *SMAD3*, and *SMAD5*) [22, 23] were specifically upregulated in C2 (Additional file 1: Fig. S1c). Genes encoding the identity of the mitochondrial respiratory chain (MRC) or C-respirasome (*COX7A1* and *COX7A2*) [24] and genes encoding subunits of mitochondrial respiratory chain complex I (*NDUFA1*, *NDUFA2*, *NDUFA3*, *NDUFB1*, and *NDUFC1*) were highly abundant in C3 (Additional file 1: Fig. S1c). In C4, genes encoding chemokines (*CCL2*, *CCL3*, and *CXCL2*), proinflammatory cytokines [*IL1A*, *IL1B*, and *TNF* (*TNFA*)] and the inflammasome (*NLRP3*) showed significantly high expression (Additional file 1: Fig. S1c). Representative signature genes of each subtype are visualized in a violin diagram (Fig. 1e). Overall, these results indicate that LDD can be categorized into 4 subtypes with different biological processes.

To externally validate the identified subtypes of LDD, we optimized gene selection by evaluating the mean decrease in accuracy and Gini coefficient of DEGs. The optimal 248-gene set comprising subtype-specific DEGs (44 for C1, 15 for C2, 19 for C3, and 170 for C4) showed the minimal error in 10-fold cross-validation (Additional file 1: Fig. S1d; Additional file 3), and was therefore selected to build an RF-based gene classifier, ensuring robust subtype discrimination. The variable importance results showed high similarity among the top 30 genes in the 248-gene classifier (Additional file 1: Fig. S1e, f). In the discovery cohort, the 248-gene classifier allocated 122 NP samples to each of the 4 subtypes with a global precision of 94.3% (115/122), resulting in AUROCs of 0.945, 0.952, 1, and 1 for C1, C2, C3, and C4, respectively, and excellent APs of 0.917, 0.935, 1, and 1 for C1, C2, C3, and C4, respectively (Additional file 1: Fig. S1g). Subsequently, to assess the generalizability of this gene classifier, we applied it to 3 independent microarray datasets from Gene Expression Omnibus (GEO) database representing 62 IVD samples from 62 donors (GSE70362, GSE23130, and GSE15227). We validated the classifier and confirmed that 24 samples from GSE70362 were assigned to C1 (79%, 19/24), C2 (17%, 4/24), and C3 (4%, 1/24), 23 samples from GSE23130 were assigned to C3 (100%, 23/23), and 15 samples from GSE15227 were assigned to C4 (100%, 15/15) (Additional file 1: Fig. S1h). These results supported the predictive potential of the 248-gene classifier and the generalizability of the molecular classification. To further validate the transcriptomic differences revealed by bulk RNA-seq analysis among clusters, we performed quantitative immunohistochemistry (IHC) for selected proteins, including GFRA1, DLX5, COX7A1, and IL-1β. Consistent with the bulk RNA-seq data, semi-quantitative IHC confirmed significantly elevated protein expression of GFRA1 in C1, DLX5 in C2, COX7A1 in C3, and IL-1β in C4 (*P* < 0.0001) (Fig. 1f). Taken together, these results indicate that 4 molecular subtypes of LDD with different biological processes were identified and that this molecular classification of LDD might be generalizable.

### Matrisome characteristics of subtype-specific ECM dysregulation traits

**Fig. 2:**
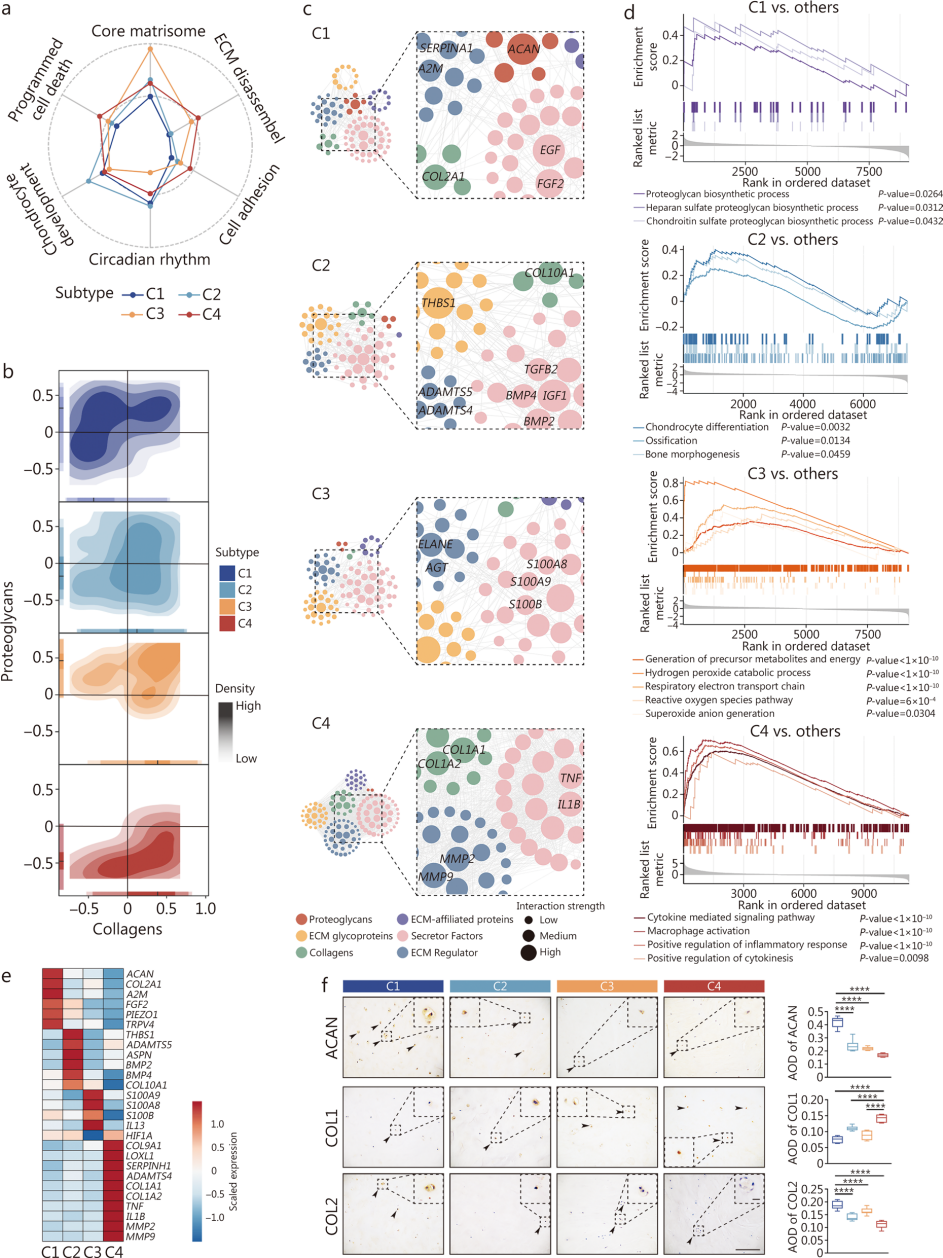
Delineating subtype-specific matrisome dysregulation traits. **a** Radar map showing the performance of 6 gene sets associated with LDD. **b** Contour map showing scores of the core ECM collagens and proteoglycans. **c** Regulatory network of upregulated DEMGs. Nodes represent upregulated DEMGs. The edge between 2 nodes represents a potential interaction. Red indicates proteoglycans, green indicates collagens, yellow indicates ECM glycoproteins, purple indicates ECM-affiliated proteins, pink indicates secreted factors, and blue indicates ECM regulators. **d** Enrichment plots of the ECM-associated gene set. The line chart indicates differences between subtypes in the individual ECM-associated gene set. **e** Heatmap showing representative subtype-specific DEMGs. **f** Representative immunohistochemical analysis of the core matrisome proteins, including ACAN, collagen I (COL1), and collagen II (COL2). Scale bar = 100 μm, insert panel = 10 μm. ^**^P < 0.01, ^****^P < 0.0001. ACAN agrrecan, AOD average optical density, C1 Cluster 1, C2 Cluster 2, C3 Cluster 3, C4 Cluster 4, COL1A1 collagen type I alpha 1 chain, COL2A1 collagen type II alpha 1 chain, DEMGs differentially expressed matrisome genes, ECM extracellular matrix, LDD lumbar disc degeneration

To better understand the specific characteristics of degenerated discs, we evaluated 4 clusters using several gene sets illustrating biological processes related to disc degeneration (Fig. 2a). The results revealed that the core matrisome significantly differed among the clusters, highlighting the existence of different ECM dysregulation patterns. Next, the core ECM structure scores for proteoglycans and collagens revealed that proteoglycans dominated in C1 and that collagens were enriched in C4 (Fig. 2b), further indicating distinct ECM composition and structure among the clusters.

Matrisome was categorized into the core matrisome (collagens, proteoglycans, and ECM glycoproteins) and the associated matrisome (ECM-affiliated proteins, ECM regulators, and secreted factors) [25]. Given that ECM composition and structure govern IVD homeostasis, we speculted that subtype-specific ECM protein aggregation and degradation might contribute to distinct mechanical and chemical microenvironments. To investgate this, we first performed comprehensive profiling of matrisome gene expression across all samples (Additional file 1: Fig. S2a), and then identified subtype-specific differentially expressed matrisome genes (DEMGs) by intersecting subtype DEGs with matrisome genes (Additional file 1: Fig. S2b, c). Subsequently, we mapped the interaction network of upregulated DEMGs (Fig. 2c). The results revealed that the DEMGs of the 6 matrisome subcategories and their potential interactions were distinct among the clusters, reflecting a unique regulatory pattern per subtype. These patterns might contribute to the subtype-specific mechanical and biochemical microenvironments of the IVD and the different stages of disc degeneration.

In C1, DEMGs encoding proteoglycans and collagens were predicted to directly interact with DEMGs encoding other matrisome subcategories (Fig. 2c). Gene set enrichment analysis (GSEA) revealed that proteoglycan biosynthesis-related terms were enriched (Fig. 2d). These results suggest that C1 represents mild degeneration. Additionally, genes encoding ECM core components (*ACAN* and *COL2A1*) were highly expressed, and the cell vitality promoter (*SERPINA1*) [26] and growth factors promoting chondrocyte proliferation (*EGF* and *FGF2*) [27] presented similar high expression patterns (Fig. 2c, e). The expression of mechanosensors (*PIEZO1* and *TRPV4*) was also strongly increased, which could enhance ECM synthesis (mainly collagen II) to sustain mechanical loading [28, 29] (Fig. 2e). This collagen II-dominated ECM remodeling in response to mechanical loading was considered to indicate collagenesis, suggesting potential mechanical adaptive repair of LDD.

In C2, the ECM interactome exhibited among DEMGs encoding ECM glycoproteins, secreted factors, and ECM regulators (Fig. 2c). However, DEMGs encoding proteoglycans and collagens were not abundant. These results suggest that ECM degradation might lead to structural disc failure. The GSEA scores also indirectly illustrated the potential for structural repair of discs via bone morphogenesis and chondrocyte differentiation (Fig. 2d). In addition, *ADAMTS5* upregulation accelerated proteoglycan degradation, and *THBS1* upregulation triggered the TGF-β1/Smad3 signaling pathway and promoted ECM remodeling in response to mechanical stress [30], whereas *ASPN* upregulation promoted collagen mineralization [31] (Fig. 2e**).** In particular, *TGFB2*, *BMP2*, *BMP4*, *IGF1*, and *COL10A1* were upregulated to promote bone morphogenesis and chondrocyte hypertrophy [23] (Fig. 2c, e). Overall, the C2 subtype might present with structural failure of the ECM, and endochondral ossification might be activated to repair the disc and stabilize the functional spinal unit.

In C3, the abundance of interacting DEMGs was minimal, with ECM glycoproteins, ECM regulators and secreted factors mainly serving as the ECM interactome, whereas the abundance of collagens and proteoglycans was significantly decreased (Fig. 2c). These results suggest that the ECM structure of C3 becomes dysfunctional. However, the significant enrichment of oxidative energy metabolism-related terms (Fig. 2d) correlated with C-respirasome activation (Fig. 1e), potentially enhancing ECM integrity and mechanostability in cartilage [32]. These results suggest that the C-respirasome was activated to adapt to oxidative stress, because the C-respirasome was more bioenergetically efficient under oxidative conditions [24]. The downregulation of *HIF1A* (Fig. 2e), a transcription factor that regulates cell behavior and viability in disc degeneration [33], also supported the oxidative microenvironment. Additionally, genes mediating inflammation and immunity (*S100A8*, *S100A9*, and *S100B*) (Fig. 2e**)**, especially *S100B*, were found to play fundamental roles in the spatial and temporal regulation of chondrogenesis [34, 35]. Unfortunately, a fewer chondrogenic genes were highly expressed in C3 (Fig. 2e). C3-subtype discs might exhibit an adverse oxidative microenvironment that inhibits chondrogenesis, resulting in severe structural damage.

In C4, collagens rather than proteoglycans were significantly enriched and interacted with other modules, forming abundant potential interactions (Fig. 2c). These results suggest that in C4, the ECM was remodeled via fibrogenesis. Inflammation and immune response-related biological processes (Fig. 2d) indicated that LDD patients experienced a severe inflammatory cascade. In addition, fibrosis markers (*COL1A1*, *COL1A2*, *TNF*, *IL1B*, *MMP2*, and *MMP9*), especially *COL1A1* and *COL1A2*, contributed to the fibrotic phenotype (Fig. 2e). *ADAMTS4*, *MMP2*, and *MMP9* are involved in ECM degradation, a process regulated by *IL1B* [36]. Notably, *TNF* and *IL1B* increase *ADAMTS4* expression to degrade aggrecan and upregulate the expression of *COL1A1* [36, 37], potentially promoting fibrogenesis. These results suggest that the inflammatory environment might drive fibrogenesis in C4-subtype discs.

Immunostaining confirmed the representative ECM protein expression among the 4 subtypes: C1 showed a significantly higher aggrecan (ACAN, *P* < 0.0001) and collagen II (COL2, *P* < 0.01) levels, whereas C4 exhibited elevated collagen I (COL1, *P* < 0.0001) (Fig. 2f). Taken together, these results indicate that the 4 subtypes exhibit distinct ECM dysregulation patterns, suggesting subtype-specific ECM remodeling in LDD.

### Subtype-specific cell subpopulations dominated the ECM phenotype per subtype

**Fig. 3:**
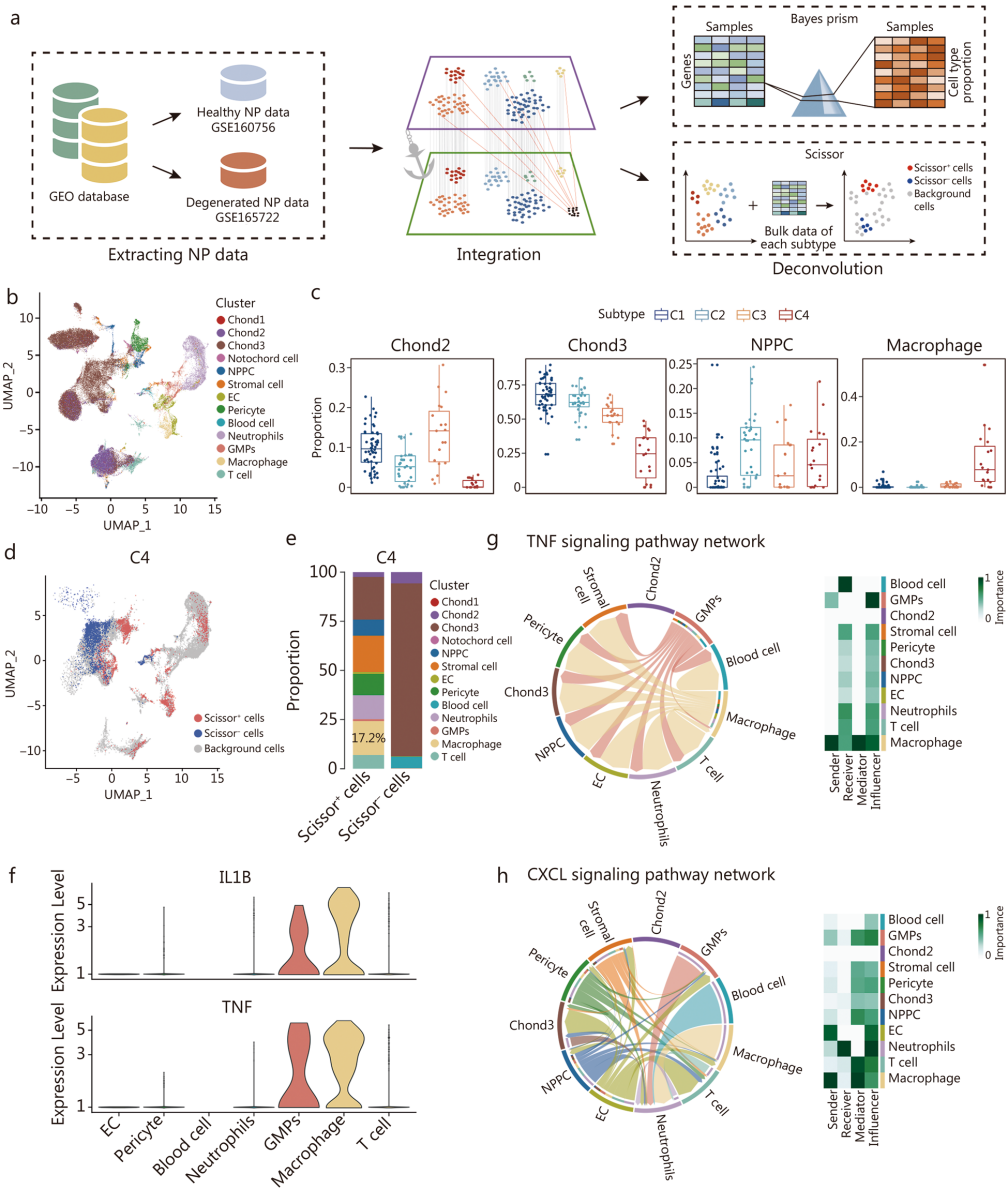
Deconvolution analysis revealing the cell subpopulations in each subtype. **a** The integration scheme of the scRNA-seq data and the deconvolution scheme of the scRNA-seq and bulk RNA-seq data with BayesPrism and Scissor. **b** UMAP visualization displaying the cell subpopulations in the integrated scRNA-seq dataset. **c** Bar chart showing the cell subpopulation proportions per subtype using BayesPrism. **d** UMAP visualization of the Scissor selected cells of the C4 subtype. The red and blue dots represent cells associated with the C4 and non-C4 subtypes, respectively. **e** Bar chart showing the cell subpopulation composition of the C4 subtype. **f** Violin plots showing the expression levels of *IL1β* and *TNF* in each cell subpopulation. Circos plot showing the TNF signaling pathway network (**g)** and the CXCL signaling pathway network (**h)** between cell subpopulations. C1 cluster 1, C2 cluster 2, C3 cluster 3, C4 cluster 4, CXCL C-X-C motif chemokine ligand, EC endothelial cells, GMPs granulocyte monocyte progenitors, NP nucleus pulposus, NPPC nucleus pulposus progenitor cell, TNF tumor necrosis factor, UMAP uniform manifold approximation and projection

Given that bulk RNA-seq can provide an overview of NP biological differences in the process of disc degeneration, it obscures the intricacies of changes within and across cell types and cannot reflect the functions of individual cell subsets. To determine the cell subpopulation composition of each subtype and explore their biological differences, two publicly available single-cell RNA-seq (scRNA-seq) datasets of IVD samples were retrieved from GEO database (GSE160756 and GSE165722) for data integration and analysis (Fig. 3a). A single-cell reference matrix of degenerated NP samples was identified by referencing our published NPC markers [38] and other published immune cell markers [39], which revealed relatively comprehensive cellular populations in the degenerated NPs (Fig. 3a, b; Additional file 1: Fig. S3a, b). Subsequently, BayesPrism [40] was employed to integrate a single-cell reference matrix of degenerated NP and the bulk RNA matrix. The results revealed that Chond2, Chond3, and NP progenitor cells (NPPCs) were highly abundant in C3, C1, and C2, respectively. Notably, C4 had a significantly high macrophage content (Fig. 3c). A relatively higher fraction of macrophages was also found in C4 by the CIBERSORT algorithm with the leukocyte signature matrix (LM22) gene signature [41] (Additional file 1: Fig. S3c). To investigate whether a high cell content is related to the ECM phenotype per subtype, we visualized the aforementioned DEMGs expression landscape of each subtype in all the cell subpopulations and found that Chond1-3 presented a relatively high abundance of *ACAN* and *COL2A1*, which contribute to the ECM phenotype in C1. In parallel, *TRPV4* and *PIEZO1* were upregulated in Chond2, further suggesting that Chond2 enhances ECM synthesis to adapt to biomechanical changes (Additional file 1: Fig. S3d)*.* Notably, Chond3, with high *SERPINE1* expression, was the dominant contributor to the antiapoptotic effect and cell viability (Additional file 1: Fig. S3d). Bone morphogenesis-related genes (*TGFB2*, *BMP4*, and *THBS1*) were enriched in NPPCs (Additional file 1: Fig. S3d), suggesting that NPPCs undergo osteogenic differentiation during the progression of disc degeneration in C2. C-respirasome signature molecules *COX7A1* and *COX7A2* [24] were expressed primarily in Chond1, with downregulation of *HIF1A* (Additional file 1: Fig. S3d), further indicating that Chond1 is involved in the hypoxic microenvironment dysregulation in C3. Surprisingly, *COL1A1* and *COL1A2* were especially expressed in stromal cells, which might contribute to the fibrotic remodeling in C4 (Additional file 1: Fig. S3d). Notably, NPPCs also expressed relatively high levels of *COL1A1* and *COL1A2*, suggesting that NPPCs in C4 presented a fibrotic phenotype.

By utilizing Scissor [42], we further revealed a high percentage of macrophages (17.2%) in C4 (Fig. 3d, e). The high expression of *IL1B* and *TNF* in macrophages (Fig. 3f) suggested inflammation and an immune response in C4. To elucidate the potential role of macrophages, we performed CellChat analysis, and the results revealed that macrophages were the dominant influencer in the TNF signaling pathway and interacted with NPPCs and Chond3 (Fig. 3g). Moreover, the C-X-C motif chemokine ligand (CXCL) signaling pathway network also revealed interactions between NPPCs and Chond3 (Fig. 3h). These intercellular interactions likely promote fibrogenesis in the NP tissues of C4. Similarly, Scissor-based prediction for C1, C2, and C3 revealed that the dominant cell subpopulation of each subtype contributed to the subtype-specific ECM dysregulation patterns (Additional file 1: Fig. S3e), which was in line with the above results (Fig. 2d). Taken together, each subtype presented distinct and unique cell subpopulations contributing to the subtype-specific ECM dysregulation phenotypes.

### Interpretable machine learning-based diagnostic prediction models that stratify molecular subtypes on the basis of clinical features

**Fig. 4:**
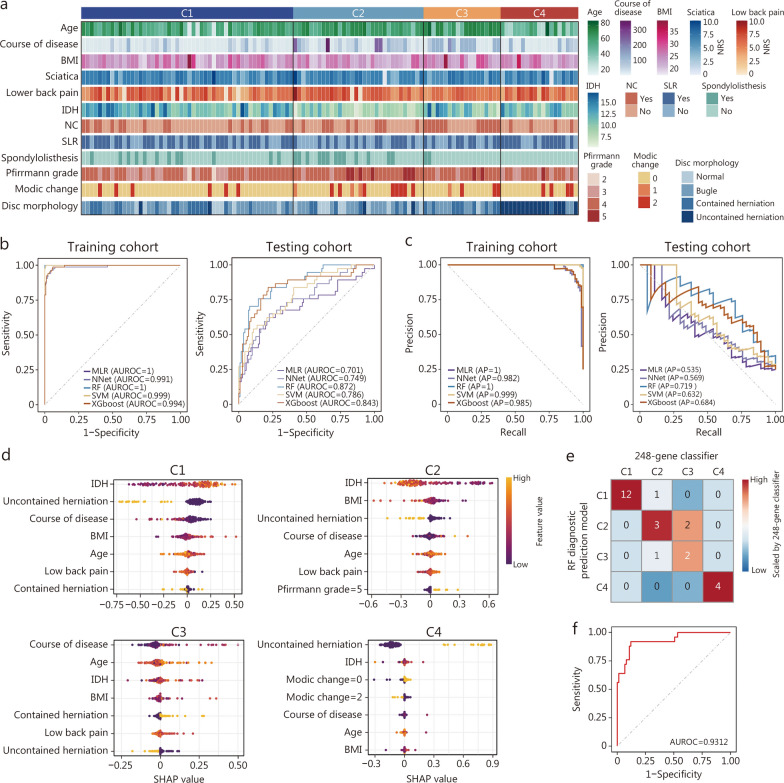
Comprehensive analysis of the machine learning-based diagnostic prediction model. **a** Heatmap representing the clinical features grouped according to the proposed LDD molecular subtypes. ROC curves and AUROCs (**b)**, PR curves and APs (**c)** derived from the training and testing sets in the discovery cohort. **d** Beeswarm visualizing attributes of the 12 most important features of the random forest predictive model in SHAP. Each line represents a feature, and the abscissa is the SHAP value. Red dots represent higher eigenvalues, and blue dots represent lower eigenvalues. Confusion matrix (**e)** and ROC curve (**f)** for testing the accuracy and AUROC of the selected RF model in the validation cohort. AP area under the PR curve, AUROC area under the ROC curve, BMI body mass index, C1 cluster 1, C2 cluster 2, C3 cluster 3, C4 cluster 4, IDH intervertebral disc height, LDD lumbar disc degeneration, MLR multinomial logistic regression, NC neurogenic claudication, NNet neural network, NRS numerical rating scale, PR precision‒recall, RF random forest, ROC receiver operating characteristic, SHAP Shapley additive explanation, SLR straight-leg-raising, SVM support vector machine

Diagnosing the molecular subtypes of LDD at the bedside remains challenging because of the potential impairment caused by disc puncture. Thus, we investigated whether a machine learning-based diagnostic prediction model could be developed on the basis of clinical features to predict molecular subtypes in clinical scenarios. First, the LASSO regression analysis was conducted to reduce the aforementioned 23 independent variables to 12 (Additional file 1: Fig. S4a), including age, body mass index (BMI), course of disease, numerical rating scale (NRS) score of low back pain, NRS score of sciatica, neurogenic claudication, straight-leg-raising test, spondylolisthesis, intervertebral disc height (IDH), Pfirrmann grade, disc morphology, and Modic changes. Next, 122 samples were randomly split into a training set (70%) and a testing set (30%) to avoid overfitting. Comparisons of clinical data between the model training and testing cohorts revealed no statistically significant differences (Table 2), indicating comparability. These potential variables were assessed among subtypes (Fig. 4a; Table 3). These 4 subtypes exhibited significant differences in age, course of disease, neurogenic claudication, spondylolisthesis, IDH, Pfirmann grade, and disc morphology (*P* < 0.05). Specifically, samples in C1 and C2 were predominantly from patients aged 50–70 years. Samples in C3 were mainly from patients older than 70 years. Most samples in C4 presented with uncontained disc herniation. Five different machine learning models, RF, SVM, XGBoost, MLR, and NNet, were evaluated using 10-fold cross-validation on the basis of the selected features. The hyperparameters of each model were optimized through a grid search within the cross-validation framework. Comprehensive analysis of the classified multimodel demonstrated that the RF model was considered the optimal model, because it achieved excellent discrimination with the highest AUROC and AP (Fig. 4b, c).

**Table 2:** Comparisons of clinical features between training and testing set

Characteristics	Training set (*n* = 85)	Testing set (*n* = 37)	*P*
Sex [*n* (%)]			> 0.05
Male	51	22	
Female	34	15	
Age (year, mean ± SD)	58.3 ± 13.0	61.1 ± 14.4	> 0.05
Height (cm, mean ± SD)	164.4 ± 9.4	162.4 ± 8.6	> 0.05
Weight (kg, mean ± SD)	67.0 ± 12.5	64.1 ± 9.0	> 0.05
BMI (kg/m^2^, mean ± SD)	24.7 ± 3.5	24.5 ± 3.6	> 0.05
Course of disease (month, median)	24	12	> 0.05
NRS of low back pain (mean ± SD)	5.6 ± 1.9	5.5 ± 2.2	> 0.05
NRS of sciatica (mean ± SD)	6.5 ± 1.5	6.4 ± 1.9	> 0.05
ODI (%, mean ± SD)	45 ± 16.7	47.2 ± 15.4	> 0.05
Numbness [*n* (%)]			> 0.05
Yes	49	16	
No	36	21	
Neurogenic claudication [*n* (%)]			> 0.05
Yes	31	12	
No	54	25	
SLR test [*n* (%)]			> 0.05
Yes	50	20	
No	35	17	
Radiographic parameters			
Spondylolisthesis [*n* (%)]			> 0.05
Yes	19	8	
No	66	29	
Osteophyte [*n* (%)]			> 0.05
Yes	63	27	
No	22	10	
IDH (mm, mean ± SD)	11.1 ± 2.7	10.6 ± 2.8	> 0.05
LL (°, mean ± SD)	36.9 ± 12.8	35.1 ± 17.3	> 0.05
SLL (°, mean ± SD)	6.2 ± 4.7	7.0 ± 4.7	> 0.05
Pfirrmann grade** [***n* (%**)]**			> 0.05
II	2	0	
III	18	8	
IV	59	25	
V	6	4	
Disc morphology [*n* (%)]			> 0.05
Normal	14	5	
Bulge	36	15	
Contained herniation	20	12	
Uncontained herniation	15	5	
Modic change [*n* (%)]			> 0.05
Normal	69	28	
I	2	2	
II	14	7	
Schmorl’s node [*n* (%)]			> 0.05
Yes	8	2	
No	77	35	

*SD* standard deviation, *BMI* body mass index, *NRS* numerical rating scale, *ODI* Oswestry disability index, *SLR* straight-leg-raising, *IDH* intervertebral disc height, *LL* lumbar lordosis, *SLL* segmental lumbar lordosis

**Table 3:** Comparisons of clinical features among four subtypes

Characteristics	C1 (*n* = 52)	C2 (*n* = 32)	C3 (*n* = 19)	C4 (*n* = 19)	*P*
Age (year, mean ± SD)	57.5 ± 13.3	63.5 ± 8.7	65.8 ± 12.9	49.8 ± 15.2	< 0.001
Age group					< 0.05
< 30	4	0	0	1	
30 **–** 40	0	0	1	5	
40 **–** 50	5	3	3	5	
50 **–** 60	20	8	1	4	
60 **–** 70	15	12	2	2	
≥ 70	8	9	12	2	
BMI (kg/m^2^, mean ± SD)	24.9 ± 3.6	23.7 ± 2.7	25.4 ± 4.5	24.8 ± 3.5	> 0.05
Course of disease (month, median)	6	36	120	4	< 0.001
NRS of lower back pain, (mean ± SD)	5.3 ± 2.3	6.0 ± 2.1	5.5 ± 0.9	5.7 ± 1.8	> 0.05
NRS of sciatica, (mean ± SD)	6.7 ± 1.4	6.4 ± 1.8	6.6 ± 1.2	6.2 ± 2.0	> 0.05
Neurogenic claudication [*n* (%)]					< 0.05
Yes	17	10	12	4	
No	35	22	7	15	
SLR test [*n* (%)]					> 0.05
Yes	33	14	10	13	
No	19	18	9	6	
Spondylolisthesis [*n* (%)]					< 0.05
Yes	12	13	2	0	
No	40	19	17	19	
IDH (mm, mean ± SD)	12.2 ± 2.4	8.8 ± 1.8	10.9 ± 2.9	11.1 ± 2.3	< 0.001
Osteophyte					> 0.05
Yes	33	28	16	13	
No	19	4	3	6	
Modic change [*n* (%)]					> 0.05
Normal	46	21	15	15	
I	0	3	1	0	
II	6	8	3	4	
Pfirmann grade** [***n* (%)]					< 0.05
II	2	0	0	0	
III	15	2	2	7	
IV	35	23	15	11	
V	0	7	2	1	
Disc morphology [*n* (%)]					< 0.001
Normal	8	10	1	0	
Bulge	26	14	9	2	
Contained herniation	16	6	9	1	
Uncontained herniation	2	2	0	16	

*SD* standard deviation, *BMI* body mass index, *NRS* numerical rating scale, *SLR* straight-leg-raising, *IDH* intervertebral disc height

To identify whether the RF model was clinically useful for this 122-sample cohort, the original 4-class molecular subtype was restructured into a set of binary classification problems, each corresponding to a different class against all others. DCA suggested that this RF model provided significant clinical benefit for identifying the C1–C4 subtypes, with probability thresholds of 5–64%, 0–87%, 9–80%, and 0–78% for each subtype, respectively (Additional file 1: Fig. S4b). To visually explain the selected features in the RF model, SHAP was utilized to illustrate how these features stratify discs into different molecular subtypes. Attributes of the most important features for each subtype in the RF model are shown in Fig. 4d and Additional file 1: Fig. S4c. These results revealed that a high IDH, bulge morphology, short course of disease, and high BMI positively contribute to C1. Interestingly, the high IDH in C1 aligned with the high expression of *ACAN* and *COL2A1* (Fig. 2e). A low IDH positively contributed to C2, followed by a low BMI and Pfirrmann grade V. A long course of disease, advanced age and contained herniation positively contributed to C3. Uncontained disc herniation, which triggers an inflammatory foreign body response [3], was a positive contributor to C4, consistent with the high expression of *TNF* and *IL1B*.

Additionally, intersubtype comparisons of the 12 clinical features provided significant information for further understanding the associations between molecular subtypes and clinical features (Table 3). Patients in the C2 and C3 groups were significantly older than those in the C1 and C4 groups. A long course of disease (median: 120 months) was observed for C3-subtype patients, highlighting that the hypoxic microenvironmental dysregulation was not achieved overnight but rather was the result of prolonged disease progression. Patients in the C1 group had excellent IDH retention while those in the C2, C3, and C4 groups presented with IDH loss, which aligned with the low expression of *ACAN* and *COL2A1*, supporting ECM collagenesis remodeling in C1. Thus, we speculate that extended conservative treatment may potentially benefit patients in the C1 group. Nearly half of the C2 patients presented with spondylolisthesis, significant IDH loss and Pfirrmann grade V degeneration (Table 3). Given that spondylolisthesis is an indicator of spinal instability [43], IDH recovery and spinal stability reconstruction may be the top priorities of surgical intervention for these patients. Most C3 patients presented neurogenic claudication (Table 3), the typical clinical presentation of spinal stenosis, indicating ischemic injury or mechanical compression of nerve roots [44]. This means that surgical intervention may be necessary for C3 patients rather than biotherapy as an initial approach. The C4 subtype is characterized by uncontained herniation triggering the inflammatory cascade, which is a double-edged sword that promotes herniated material resorption and causes pain [3]. Thus, managing inflammation may be the core challenge of treating patients in the C4 group. Moreover, to illustrate the interpretability of the RF model, a waterfall plot was used to visualize the impact of features in a typical example from each subtype on the model output. The SHAP values for typical patients from the C1, C2, C3, and C4 groups were 0.906, 0.972, 0.976, and 0.996, respectively (Additional file 1: Fig. S4d). Collectively, the attributes of the clinical features could explain the stratification of each patient.

To examine the accuracy of the RF model in stratifying LDD molecular subtypes, 25 NP samples from 25 LDD patients were collected at Army Medical Center of PLA and used as an independent validation cohort (Additional file 2: Table S3). On the basis of the bulk RNA matrix, the 248-gene classifier, the gold standard for LDD molecular classification, assigned 12, 5, 4, and 4 samples to the C1, C2, C3, and C4 subtypes, respectively. The RF model leveraging 12 clinical features stratified 13, 5, 3, and 4 samples to the C1, C2, C3, and C4 subtypes, respectively (Fig. 4e). Confusion matrix and ROC analyses revealed that the RF model identified LDD molecular subtypes with an accuracy of 0.84 and an AUROC of 0.9312 (Fig. 4e, f). The RF model performed robustly in an independent cohort, indicating that the RF model is a clinically applicable tool for LDD patient stratification and diagnosis. Taken together, the RF model-based association between molecular classification and clinical features holds significant potential for guiding the treatment of LDD patients in the clinical setting.

### Macrophages dominate inflammation-induced fibrosis in C4-subtype discs

**Fig. 5:**
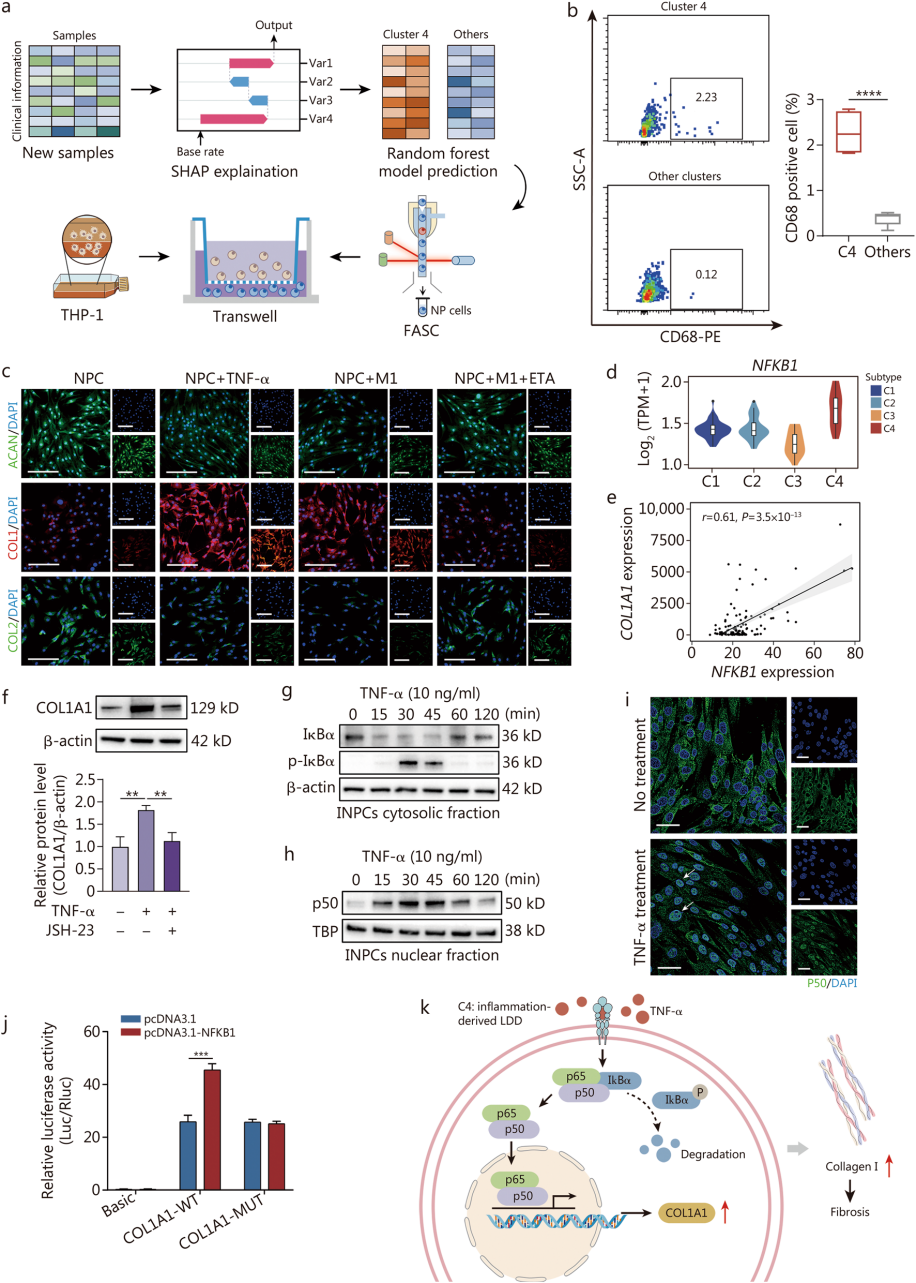
NPC‒M1 macrophage interactions contribute to NPC fibrotic phenotype ex vivo, and TNF-α influences COL1A1 expression in INPCs via the transcription factor NF-κB1 (p50) in vitro. **a** Scheme of RF-based LDD subtype prediction, flow cytometry analysis of CD235a^−^CD31^−^CD68^+^ cells and CD235a^−^CD31^−^CD68^−^CD45^−^ NPC sorting, and transwell coculture of NPCs and M1 macrophages differentiated from THP-1 monocyte lines. **b** Representative flow cytometry isolation of CD235a^−^CD31^−^CD68^+^ cells from NP tissues. **c** Representative immunofluorescence analysis of selected core ECM protein (ACAN, collagen I (COL1), and collagen II (COL2)) via a transwell assay. Scale bar = 100 μm. **d** Violin plots showing significant upregulation of *NFKB1* in C4. **e** Pearson correlation analysis of *COL1A1* with *NF-κB1* in the bulk RNA-seq dataset. **f** Immunoblot and densitometry plots (*n* = 3) of COL1A1 in INPCs after treatment with TNF-α (10 ng/ml) or JSH-23 (10 μmol/L) for 24 h. Immunoblots showing the time-dependent expression of IκBα and p-IkBα in the cytosolic extracts (**g)** and NF-κB1 (p50) in the nuclear extracts (**h)** of INPCs treated with TNF-α (10 ng/ml) for 24 h. **i** Immunofluorescence analysis of INPCs treated with TNF-α (10 ng/ml) for 30 min and stained for p50 (green) and nuclei (blue). The arrows show the nuclear localization of p50. Scale bar = 50 μm. **j** Fluorescence activity in 293T cells with wild-type and mutant COL1A1 promotors with pcDNA3.1-NFKB1. **k** Schematic graph showing that TNF-α-induced p50 activation enhances COL1A1 expression. ^*^P < 0.05, ^**^P < 0.01, ^***^P < 0.001, ^****^P < 0.0001. ACAN aggrecan, C1 cluster 1, C2 cluster 2, C3 cluster 3, C4 cluster 4, COL1A1 collagen type I alpha 1 chain, ECM extracellular matrix, ETA etanercept, FASC fluorescence-activated cell sorting, INPCs immortalized NP cells, LDD lumbar disc degeneration, MUT mutant, NF-κB1 nuclear factor kappa B subunit 1, NP nucleus pulposus, NPC nucleus pulposus cell, RF random forest, SHAP Shapley additive explanation, TBP TATA binding protein, TNF-α tumor necrosis factor-α, WT wide-type

Considering that the macrophage-rich C4 subtype could be predicted using the proposed RF model, ten NP samples were collected at Army Medical Center of PLA and used as another independent cohort. The RF model was used to predict the molecular subtypes of these samples based on the clinical features of the patients (Additional file 4), followed by single-cell extraction. CD235a^−^CD31^−^CD68^+^ macrophages were examined by fluorescence-activated cell sorting, and CD235a^−^CD31^−^CD68^−^CD45^−^ NPCs were sorted for passage culture (Fig. 5a). As expected, the proportion of CD235a^-^CD31^-^CD68^+^ macrophages in C4 was significantly higher than that in other subtypes (Fig. 5b), which aligns with prior studies indicating a 3-fold increase of CD68^+^ macrophages in uncontained disc herniation [45].

To validate the ECM phenotype of M1 macrophage-NPC interactions, THP-1 (ATCC TIB-202, Manassas, VA)-derived M1 macrophages were indirectly cocultured with P2 CD235a^−^CD31^−^CD68^−^CD45^−^ NPCs from C4 samples (Fig. 5a). The results revealed that TNF-α (10 ng/ml)-treated NPCs upregulated collagen I expression, whereas the expression of aggrecan and collagen II was significantly decreased. In parallel, NPCs cocultured with M1 macrophages showed similar expression patterns of collagen I, collagen II and aggrecan; moreover, these interaction effects were partially reversed by the TNF-α inhibitor etanercept (10 ng/ml, HY-108847, MCE) (Fig. 5c; Additional file 1: Fig. S5a). Collectively, these findings indicate that macrophage-secreted TNF-α contributes to COL1A1 upregulation.

We further investigated the mechanism of TNF-α regulation of COL1A1. Immortalized NP cells (INPCs) (iCELL-0028a, iCell Bioscience Inc., Shanghai) were exposed to varying doses of TNF-α. The results revealed a significant increase in COL1A1 expression at both the mRNA and protein levels with 10 ng/ml TNF-α treatment (*P* < 0.001; Additional file 1: Fig. S5b-d), which aligns with previous findings [37]. Interestingly, *NFKB1* was significantly upregulated in C4 (Fig. 5d), whereas other fibrosis-related transcription factors (*JUN*, *SP1*, *TGFB1*, and *SMAD3*) did not show significant changes in C4 (Additional file 1: Fig. S5e). Furthermore, a positive correlation was confirmed between *COL1A1* and *NFKB1* (*r* = 0.61, *P* < 0.001; Fig. 5e) but not between *COL1A1* and *JUN*, *SP1*, *TGFB1*, or *SMAD3* (Additional file 1: Fig. S5f). These results suggested that targeting inflammation might have potential benefits for ameliorating fibrosis of C4. We hypothesized that TNF-α might induce NF-κB1 (p50) activation to increase COL1A1 expression. Subsequent treatment of INPCs with 10 ng/ml TNF-α and 10 μmol/L JSH-23 (a selective inhibitor of NF-κB signaling) significantly reduced COL1A1 expression in the JSH-23-pretreated group (10 μmol/L) (*P* < 0.01; Fig. 5f), demonstrating the impact of NF-κB activation on COL1A1 expression.

In unstimulated cells, NF-κB remains inactive when bound to inhibitors of κB (IκB, IκBα, and IκBβ) in the cytoplasm. Treatment of INPCs with TNF-α resulted in rapid degradation of IκBα within 30 min and a concomitant increase in cytosolic p-IκBα and nuclear NF-κB1 (p50) levels (Fig. 5g, h; Additional file 1: Fig. S5g, h). Immunostaining confirmed NF-κB1 (p50) sequestration in the nucleus after 30 min of TNF-α treatment (Fig. 5i). Thus, TNF-α induces IκBα degradation in INPCs and facilitates rapid translocation of NF-κB1 (p50) to the nucleus, a hallmark of NF-κB signaling activation. Subsequently, luciferase reporter vectors containing wild-type and mutant (MUT) NF-κB1 (p50) binding sequences of COL1A1 were constructed. Cotransfection of the COL1A1 promoter fragment with the NF-κB1 (p50)-expressing vector significantly increased the luciferase activity compared with that of the control (the COL1A1 promoter fragment cotransfected with the empty vector) (*P* < 0.001; Fig. 5j). Conversely, cotransfection of the NF-κB1 (p50)-expressing vector with the COL1A1-MUT construct led to a significant decrease in luciferase activity over the former (*P* < 0.001) but did not affect luciferase activity compared with the control (Fig. 5j). These findings suggest that NF-κB1 (p50) positively modulates COL1A1 promoter activity (Fig. 5k). Overall, TNF-α-induced activation of NF-κB1 (p50) leads to the upregulation of COL1A1 expression.

## Discussion

This study addresses significant challenges in translating biotherapy for LDD into clinical practice, primarily stemming from limited understanding of the pathology and molecular complexity of LDD, as well as difficulties in effective patient stratification and diagnosis [8, 9]. To address these issues, we developed a comprehensive molecular classification that categorizes LDD into 4 distinct subtypes (C1–C4). Specifically, C1 showed elevated expression of *COL2A1* and mechanosensors, including *PIEZO1* and *TRPV4* (Fig. 2), which could increase collagen II synthesis to sustain mechanical loading [28, 29], thus identifying it as load-stressed LDD. In contrast, C2 exhibited signatures associated with bone morphogenesis and chondrocyte hypertrophy (Fig. 2), suggesting ossification-mediated repair, leading to its classification as ossification-activated LDD. Meanwhile, the harsh oxidative microenvironment in C3 likely impaired chondrogenesis (Fig. 2), supporting its designation as chondrogenesis-limited LDD. Finally, C4 exhibited a high level of inflammatory response signatures (*IL1B* and *TNF*), which have been shown to upregulate collagen I expression (Figs. 2 and 5), designating this subtype as inflammation-derived LDD. This classification not only provides a detailed molecular landscape of LDD but also reveals subtype-specific ECM metabolic characteristics and pinpoints potential molecular targets of therapeutic intervention for each subtype.

Notably, subtype heterogeneity existed among discs from different levels of the same LDD patient (Additional file 2: Table S2), indicating that the same biotherapeutic strategy cannot be generalized to all degenerated discs in the same patients. By integrating bulk RNA-seq and scRNA-seq data, we highlighted the dominant cell subpopulation states within each subtype, providing insights into the cellular diversity present in LDD. Additionally, by utilizing an RF model based on clinical features, we established a clinically applicable framework for patient stratification and diagnosis. In brief, this study not only established a robust molecular classification for LDD but also elucidated significant associations between each subtype and distinct clinical features, laying a foundation for personalized therapeutic strategies (Figs. 1 and 4). Furthermore, we validated the fibrosis-inducing interactions between NPCs and macrophages in C4 and elucidated that TNF-α-induced NF-κB1 (p50) activation promoted COL1A1 expression (Fig. 5), emphasizing the role of inflammation in disease progression. These findings provide crucial insights into the pathology and heterogeneity of LDD, paving the way for tailored subtype-specific biotherapies that could improve patient outcomes.

In our investigation of ECM metabolic dysregulation, molecular features, and cellular composition across the 4 subtypes, we revealed correlations with clinical features (Figs. 2 and 4). By integrating bulk RNA-seq with scRNA-seq, we identified distinct matrisome profiles and key cellular populations driving ECM remodeling in each subtype. C1 is characterized by elevated *TRPV4* and *PIEZO1* expression alongside expression of chondrogenic markers such as *ACAN* and *COL2A1*, indicating early degenerative changes where ECM collagenesis acts as a compensatory repair response to mechanical stress [28, 29]. Notably, Chond1-3 within C1 play crucial roles in ECM homeostasis, as they express collagen II and aggrecan, which support tissue repair and potentially prevent nerve infiltration [38]. Along with the excellent IDH, these molecular characteristics align with a subtype of mild degeneration. In C2, elevated *ADAMTS5* expression undermines proteoglycan integrity and leads to significant IDH loss, as observed radiographically. The predominant NPPCs in C2 exhibit osteogenic features, with high expression of genes such as *BMP4*, *TGFB2*, and *THBS1*, indicating an adaptive response to spinal instability, which is often linked with spondylolisthesis [46]. Furthermore, *ASPN* expression emphasizes collagen mineralization, reflecting a structural response to instability typical in this subtype. C3 showed a metabolic shift toward oxidative phosphorylation, as evidenced by the upregulation of COX7A subunits, which likely stabilized the ECM and aligned with the dominance of Chond1 in C3 [24, 38]. Clinically, this subtype is frequently associated with lumbar spinal stenosis and neurogenic claudication, underscoring the role of metabolic adaptation in ECM degradation and symptom manifestation. C4 displayed a marked inflammatory response characterized by elevated expression of *TNF* and *IL1B*, along with high levels of *MMP2* and *MMP9*, suggesting extensive ECM dysregulation [36, 37]. Emerging evidence suggests that miRNA, lncRNA, and methylation modulate ECM remodeling by targeting IL-1β, TNF-α, MMPs, and the NF-κB pathway [47, 48]. Potential crosstalk between transcriptional and epigenetic regulation may contribute to LDD progression. The investigation of distinct epigenetic signatures across LDD subtypes could further refine the molecular classification. Undeniably, anti-inflammatory and antifibrotic therapies might mitigate disease progression, especially in case of uncontained disc herniation where inflammatory cells exacerbate tissue damage. These distinct ECM and cellular profiles provide a foundation for personalized treatment approaches. In summary, the unique ECM and cellular profiles identified across subtypes C1 to C4 establish a basis for personalized treatment strategies. Targeting subtype-specific molecular and cellular changes, such as *TRPV4* and *PIEZO1* modulation in C1, structural realignment in C2, hypoxic adaptation in C3, and TNF-α or NF-κB signaling inhibition in C4, could enhance therapeutic outcomes and drive advancements in precision medicine for LDD management.

Moreover, we employed machine learning algorithms to connect clinical features with these molecular subtypes, addressing the existing gap in LDD stratification. Although there was an imbalance between males and females, no significant difference was detected between the training and testing sets (*P* > 0.05; Table 2), and the distribution of this characteristic in the discovery cohort (Tables 1 and 2) was consistent with the known epidemiological pattern [49]. Among the various models tested, the RF model demonstrated the highest accuracy in predicting molecular subtypes on the basis of 12 clinical features. Although the RF model performed robustly (AUROC: 0.9312; accuracy: 0.84) in an independent cohort, which indicates its clinical applicability, a larger sample size in multicenter validations is needed before its clinical implementation. While this model is not intended to replace direct molecular profiling, it illustrates the feasibility of a noninvasive, clinically interpretable tool for preliminary subtype diagnosis. This approach might improve patient stratification in clinical scenarios, helping to overcome the heterogeneity that has impeded biotherapeutic research in LDD.

Traditional classification systems that focus solely on morphological or histological features fail to capture the underlying molecular heterogeneity of LDD progression. The clinical relevance of our molecular subtypes (C1 to C4) lies in their correlation with existing clinical features of LDD, suggesting new directions for precise therapeutic interventions. Specifically, the load-stressed subtype (C1) corresponds to patients who have relatively higher IDHs and mild early degeneration. Biotherapy may be optimal for C1. The ossification-activated subtype (C2) is associated with spondylolisthesis and disc narrowing on radiographic imaging, as well as potential calcification or ossification tendencies within the disc tissue, indicating that rigidity and pain are generated. The chondrogenesis-limited subtype (C3) is linked to patients exhibiting decreased proteoglycan levels, typically corresponding to disc narrowing and neurogenic claudication, indicating lumbar spinal stenosis. For the C2 and C3 subtypes, spinal alignment reconstruction should be prioritized. Emerging integrative bioengineering methodologies leveraging advanced cellular therapies and functional biomaterials may offer a transformative treatment avenue by concurrently addressing biomechanical integrity and biological repair. Particularly noteworthy is the inflammation-derived subtype (C4), which is associated with severe lumbar disc herniation; approximately 85% of C4 patients demonstrate uncontained herniations, a condition characterized by pronounced inflammatory responses and fibrosis. This distinct clinical-pathological correlation prompted our investigation into the pathological mechanism of C4 subtype discs, revealing NF-κB-mediated fibrotic remodeling as a central pathological cascade. These findings highlight anti-inflammatory or immunomodulatory therapies as potential therapeutic avenues.

Although this is the first study to establish the clinical-transcriptomic molecular classification of LDD on the basis of a large cohort, it has several limitations. First, the single-center design identified an age-related predisposition to LDD, consistent with the known epidemiological pattern [49]. Nevertheless, the modest sample size currently precludes an in-depth analysis of gene expression variations across different age groups (Table 1). Second, although this study inferred the dominant cell subpopulation state of each subtype by integrative analysis, single-cell transcriptomic validation is required to definitively characterize these cellular states. Third, ethical and practical challenges in obtaining healthy human IVD specimens results in a lack of normal NP tissues for direct comparisons. Thus, the subtype-specific ECM dysregulation patterns and their proposed therapeutic targets warrant further validation.

## Conclusions

In conclusion, our integrative molecular analysis of degenerated NPs illuminates the complex molecular landscape of LDD, revealing 4 distinct subtypes with unique ECM regulatory patterns, cellular compositions, and microenvironments. Importantly, the selected RF model holds promise as a noninvasive and clinically interpretable tool, enhancing patient stratification and facilitating more personalized interventions in clinical settings. Collectively, these findings pave the way for tailored therapies for LDD and contribute to the advancement of personalized medicine in this challenging field.

## Supplementary Information

**Additional file 1.** Materials and methods. **Fig. S1** Molecular classification by unsupervised clustering and external microarray validation. **Fig. S2** Attributes of matrisome-associated genes per sample in each subtype. **Fig. S3** Subtype-specific cell subpopulations and functional phenotypes. **Fig. S4** Feature selection for the machine learning model and the clinical usability evaluation, and SHAP explanation of the RF model. **Fig. S5** TNF-α influences COL1A1 expression in INPCs in vitro.

**Additional file 2.** **Table S1** Clinical data of the discovery cohort included 122 NP tissues from 108 patients. **Table S2** Clinical data of 28 samples from 14 patients in the discovery cohort. **Table S3** Clinical data of the validation cohort included 25 NP tissues from 25 patients.

**Additional file 3.** Clustering results, DEGs of each subtype, and 248 DEGs in the gene classifier.

**Additional file 4.** Clinical data of the individual cohort included 10 NP tissues for flow cytometry sorting.

## Data Availability

All the data associated with this study are presented in the paper and the Additional files. The raw bulk RNA-seq data have been uploaded to the GSA repository (https://ngdc.cncb.ac.cn/gsa-human), and the accession code for the data is HRA009301. All other relevant data in this study are available from the corresponding authors upon reasonable request. The bulk RNA-seq data matrix used in this study is available from the online digital repository platform FigShare, and the link to access the data is 10.6084/m9.figshare.27642930.v1. The R scripts for data analysis are shared in the public code repository GitHub, and the link to permanently access the code is https://github.com/Lp3533/Code-of-IDD-classification.

## Abbreviations

AP: Area under the PR curve
AUROC: Area under the ROC curve
BMI: Body mass index
DCA: Decision curve analysis
DEGs: Differentially expressed genes
DEMGs: Differentially expressed matrisome genes
ECM: Extracellular matrix
GO: Gene Ontology
GSEA: Gene set enrichment analysis
IDH: Intervertebral disc height
IHC: Immunohistochemistry
INPC: Immortalized nucleus pulposus cell
IVD: Intervertebral disc
LASSO: Least absolute shrinkage and selection operator
LDD: Lumbar disc degeneration
MRC: Mitochondrial respiratory chain
MRI: Magnetic resonance imaging
NP: Nucleus pulposus
NPC: Nucleus pulposus cell
NPPC: Nucleus pulposus progenitor cell
NRS: Numerical rating scale
PR: Precision‒recall
RF: Random forest
ROC: Receiver operating characteristic
SC3: Single-cell consensus clustering
SHAP: Shapley additive explanation
