# Supplementary material for: Clinical-transcriptomic classification of lumbar disc degeneration enhanced by machine learning
Source: Mil Med Res. 2025 Aug 29;12:54. doi: 10.1186/s40779-025-00637-9 (PMC12395706; doi:10.1186/s40779-025-00637-9)
Supplement: Supplementary file 1 — Additional file 1. Materials and methods. Fig. S1 Molecular classification by unsupervised clustering and external microarray validation. Fig. S2 Attributes of matrisome-associated genes per sample in each subtype. Fig. S3 Subtype-specific cell subpopulations and functional phenotypes. Fig. S4 Feature selection for the machine learning model and the clinical usability evaluation, and SHAP explanation of the RF model. Fig. S5 TNF-α influences COL1A1 expression in INPCs in vitro. [file 40779_2025_637_MOESM1_ESM.pdf]

Materials and methods

Patient enrollment

Patients were consecutively recruited by invitation at Army Medical Center of PLA (Daping Hospital of Army Medical University). The discovery and validation cohorts were prospectively establised from November 20, 2022, to June 20, 2023, and from January 15, 2025, to February 28, 2025, respectively. A total of 108 patients with 122 intervertebral disc (IVD) samples were enrolled in the discovery cohort, and 25 patients with 25 IVD samples were included in the validation cohort. Inclusion criteria were as follows: i) aged 18 years or older, ii) primary presentation of low back pain and/or sciatica, and iii) requirement for discectomy and/or intervertebral fusion. Notably, IVD degeneration was confirmed in patients by lumbar magnetic resonance imaging (MRI). The exclusion criteria were: i) prior surgical intervention affecting the responsible disc, spinal tumors, or spinal infection; ii) other concomitant diseases rendering patients unsuitable for this study; and iii) participation in other studies. Informed consent for this study was obtained from all patients.

Clinical data collection

The clinical information of prospective patient cohorts was collected at the time of the initial clinical assessment. The demographic data included age, sex, height, weight, body mass index, diabetes status, smoking status, and history of previous lumbar spine or IVD surgery. The clinical characteristics included course of disease, pain location and intensity, neurogenic claudication, numbness, the Oswestry Disability Index, muscle strength, and straight-leg-raising test score. Low back pain and sciatica were evaluated by a numerical rating scale.

An MRI scan of the lumbar spine with T1-weighted and T2-weighted sequences was performed using a 1.5-Tesla system (Signa EXCITE; General Electric Health Care, United States). Preoperative

lumbar plain radiography and MRI were collected. Lumbar radiographs were used to assess spondylolisthesis and osteophytes. Radiographic parameters, including the intervertebral disc height (IDH), lumbar lordosis (LL), and segmental LL, were measured via the lateral standing lumbar radiographs. The disc morphology on the lumbar MR image was categorized as one of four subclassifications by combining the lumbar MRI and intraoperative validation.

Collection of IVD tissues

Freshly resected IVD tissues from patients were obtained, and the nucleus pulposus (NP) was identified with a microscope. Sufficient NP tissues to meet the minimum bulk RNA sequencing (RNA-seq) requirements were collected and divided into two parts, as follows: one part was stored in MACS tissue storage solution at 4 °C for primary NP cell (NPC) isolation, and the other (approximately 5 mm × 5 mm × 5 mm) was fixed in 4% paraformaldehyde within 72 h for paraffin sectioning.

Isolation of primary NPC

For single-cell NP suspensions, all samples were first washed several times with phosphate-buffered saline (PBS) to eliminate visible blood contamination, minced into small pieces (less than 3 mm) on ice, treated with 1× red blood cell lysis buffer (130-094-183, Miltenyi, Germany) at 5 times volume of tissues at 4 °C for 5 min, and then filtered through a 70-μm cell strainer. The tissue was enzymatically digested using a cocktail with 0.2% pronase (10165921001, Roche, USA), 0.2% collagenase II (C6885, Sigma-Aldrich, USA), 100 U/ml hyaluronidase (H8030, Solarbio, China), and 100 U/ml DNase I (D8071, Solarbio, China) at 37 °C in an atmosphere of 5% CO_2 for 3 h until the tissue was digested into a single-cell suspension. Gentle rotation was performed every 20 min. After digestion, the isolated cells were filtered through a 70-μm cell strainer and centrifuged at 300×*g* for 5 min. After washing with PBS, the cell fragments were removed. Dissociated NPC were then stained with acridine orange(AO) / propidium iodide (PI) for cell counting and viability assessment using a Rigel S2 Cell

Counter (Countstar, China). Cell pellets were collected for subsequent experiments.

RNA extraction and reverse transcription

After the pellets were harvested, total RNA was extracted by TRIzol reagent (B511311-0025, BBI, UK) according to the manufacturer's instructions. The RNA concentration and purity were measured by a NanoDrop 3300 (Thermo Scientific™, Germany). The Agilent 2100 Bioanalyzer (Agilent, USA) was used to assess the integrity of the RNA, and only those samples with an RNA integrity number (RIN) ≥ 7 were included in the subsequent procedures.

Library preparation and sequencing

The RNA library was constructed, and subsequent RNA sequencing was performed on the DNBSEQ platform (BGI-Shenzhen, China). SOAPnuke [1] was used to filter the raw sequencing reads; afterward, the clean reads were obtained and stored in FASTQ. The clean reads were subsequently mapped to the human reference genome (hg38) using HISAT2 [2]. Raw counts of protein-coding genes were obtained via featureCounts [3]. The aligned read counts by gene were converted into a count matrix, which was analyzed using the DESeq2 (v.1.40.2) [4] package for count normalization.

Primary NPC sorting and culture, and macrophage examination

Cell pellets were suspended in 3% bovine serum albumin (BSA) and incubated with a FITC-conjugated anti-human CD235a (glycophorin A) antibody (11-9987-82, eBioscience™, Invitrogen, USA), a FITC-conjugated anti-human CD31 antibody (303104, Biolegend, USA), a PE-Cyanine7-conjugated anti-human CD45 antibody (25-0451-81, eBioscience™, Invitrogen, USA), a PE-conjugated anti-human CD68 antibody (12-0689-42, eBioscience™, Invitrogen, USA) and the fixable viability dye eFluor™ 780 (65-0865-14, eBioscience™, Invitrogen, USA). $CD235a^{-}CD31^{-}CD68^{+}$ cells were examined. $CD235a^{-}CD31^{-}CD68^{-}CD45^{-}$ NPC were sorted using BD Arial II and cultured in standard

media consisting of DMEM/F12, 10% fetal bovine serum, and 1% penicillin/streptomycin (sv30010, HyClone, USA) in T25 cell culture flasks; the cells were cultured in a humidified incubator at 37°C under 5% CO_2. After 3 – 4 days, the medium was completely changed, and the adherent cells were cultured and proliferated by refreshing the medium every 3 d. When the cells reached about 80% confluency, the primary cells were harvested and passaged. Passage 1 (P1) cells were harvested with 0.25% trypsin ethylenediaminetetraacetic acid (SH30042.01, HyClone, USA) for subsequent coculture experiments.

Macrophage polarization induction and M1 macrophage collection

The human monocyte cell line THP-1 (ATCC TIB-202, Manassas, VA, USA) was induced to differentiate into M1 macrophages by phorbol 12-myristate 13-acetate (PMA), lipopolysaccharide (LPS), and interferon-γ (IFN-γ) stimulation as previously described [5,6]. Briefly, cells were seeded at a density of 3×10^6 in 6-cm culture dishes and treated with 100 ng/ml PMA (P1585, Sigma, USA) for 48 h to achieve M0 polarization. Next, the cells were treated with 100 ng/ml LPS (L2880, Sigma, USA) and 20 ng/ml IFN-γ (HY-P7025, MCE, China) for another 48 h to promote M1 polarization. M1 macrophages were harvested with 0.25% trypsin ethylenediaminetetraacetic acid (SH30042.01, HyClone, USA) for identification and subsequent coculture experiments.

Coculture of P2 NPC and M1 macrophages

Using a transwell (3450, Corning, USA) coculture system, M1 macrophages and NPC were indirectly cocultured at a ratio of 1:1 for 48 h. Briefly, P2 NPC were cultured at 5×10^4 cells/well in the lower chamber of a 24-well plate containing a coverslip, and M1 macrophages were plated in the upper chamber. Four groups were assigned, as follows: (1) P2 NPC were cultured alone in standard medium as a control; (2) P2 NPC were treated with 10 ng/ml tumor necrosis factor alpha (TNF-α) (SRP3093, Sigma, USA); (3) P2 NPC were cocultured with M1 macrophages that were separated in transwell

inserts; and (4) P2 NPC were indirectly cocultured with M1 macrophages and treated with 10 ng/ml etanercept (HY-108847, MCE, China). After 48 h, the coverslips were fixed in 4% paraformaldehyde for subsequent staining.

Immortalized NP cell (INPC) culture and treatment

INPC (iCELL-0028a, iCell Bioscience Inc., Shanghai, China) were cultured in DMEM/F12 supplemented with 10% fetal bovine serum (FBS, Gibco, USA) and 1% penicillin/streptomycin in a 5% CO_2 incubator. INPC were treated with 0, 1, 5, 10, 20, and 50 ng/ml TNF-α for 24 h to examine *COL1A1* expression at both the mRNA and protein levels. To investigate whether TNF-α-induced nuclear factor kappa B subunit 1 (NF-κB1) (p50) activation could regulate *COL1A1* expression, three groups were assigned: 1) INPC cultured alone, 2) INPC treated with 10 ng/ml TNF-α (SRP3093, Sigma, USA), and 3) INPC pretreated with 10 μmol/L JSH-23 (S7351, Selleck, USA), a selective inhibitor of NF-κB signaling for 30 min, and treated with 10 ng/ml TNF-α (SRP3093, Sigma, USA) for 24 h. To investigate p50 activation kinetics, INPC were treated with 10 ng/ml TNF-α for 0, 15, 30, 45, 60, or 120 min.

Protein isolation and Western blotting

After treatment, the INPC were immediately placed on ice and washed twice with ice-cold phosphate-buffered saline. The cells were harvested and lysed in RIPA buffer, and total protein concentrations were measured using a bicinchoninic acid (BCA) protein concentration assay kit (P0010S, Beyotime, China). The cytoplasmic and nuclear proteins from INPC were extracted using a nuclear and cytoplasmic protein extraction kit (P0027, Beyotime, China). Total cellular proteins (20 μg/well) were resolved via 10% sodium dodecyl sulfate-polyacrylamide gel electrophoresis and transferred to polyvinylidene difluoride membranes (0.22 μm pore; FFP26, Beyotime, China). The membranes were blocked in 5% nonfat dry milk or BSA (ST023, Beyotime, China) with Tris-buffered saline with Tween

20 (TBST) for 2 h and incubated overnight at 4 °C with primary antibodies recognizing the following proteins: collagen I (1:1000, ab260043, Abcam, UK), IκBα (1:2000, 80019-1-RR, Proteintech, USA), p-IκBα (1:1000, 2859S, CST, USA), p65 (1:1000, 8242S, CST, USA), p-p65 (1:1000, 3033S, CST, USA), and p50 (1:1000, 13586S, CST, USA). β-actin (1:1000, A8227-50, Abcam, UK) and TATA binding protein (TBP) (1:1000, 22006-1-AP, Proteintech, USA) were used as loading controls for the data analyses. After being washed with TBST, the membranes were incubated with anti-rabbit or mouse horseradish peroxidase-conjugated secondary antibodies, which were subsequently detected with enhanced chemiluminescence (34094, Thermo Fisher Scientific, USA). The results were subsequently quantified using ImageJ software.

Quantitative real-time reverse transcription-polymerase chain reaction (qRT-PCR)

Total RNA was isolated from INPC using the Fastpure cell/tissue total RNA isolation kit V2 (RC112, Vazyme, China) and reverse transcribed with HiScript Ш RT SuperMix for qPCR (+ gDNA wiper) (R323, Vazyme, China) according to the manufacturer’s protocol. Real-time PCR was run using a HiScript II ONE Step qRT–PCR SYBR Green Kit (Q221, Vazyme, China), and GAPDH was used as an internal control. The sequences of the primers used herein were as follows: COL1A1: forward (5′-GCTCGTGGAAATGATGGTGC-3'), reverse (5′-ACCCTGGGGACCTTCAGAG-3′), GAPDH: forward (5′- CTGGGCTACACTGAGCACC -3'), reverse (5′- AAGTGGTCGTTGAGGGCAATG -3′).

Dual-luciferase reporter gene assay

The 2300-base pair region upstream of *COL1A1* was selected as the promoter region on the basis of information from the National Center for Biotechnology Information database (http://www.ncbi.nlm.nih.gov/). Wild-type (WT) and mutant (MUT) *COL1A1* fragments were synthesized by Dynegene (Shanghai, China). The luciferase gene was cloned downstream of the wild-type or mutant region of *COL1A1*. A total of 1×10^5 293T cells were seeded per well in 48-well plates

and cotransfected with Lipo8000™ Transfection Reagent (C0533, Beyotime, China) and pcDNA3.1 or pcDNA3.1-NFKB1 along with pGL1-Basic, pGL1-COL1A1 promoter-WT or pGL1-COL1A1 promoter-MUT. After 72 h, the cells were lysed, and firefly luciferase activity was measured with a Dual Luciferase Reporter Assay System (RG088S, Beyotime, China) and normalized to that of Renilla luciferase.

Immunohistochemistry (IHC) assay

After routine fixation, the samples were dehydrated and embedded in paraffin. The paraffin-embedded NP samples were cut into 4-μm-thick serial sections. The sections were deparaffinized, rehydrated, and then washed three times with PBS. Antigen retrieval was performed using pepsin (ZLI-9013, ZSGB-BIO, China) for 20 min, followed by incubation with 3% H_2O_2 for 30 min. Then, the sections were blocked with 5% BSA for 30 min. The above processes were finished at room temperature. The sections were subsequently incubated with anti-glial cell line-derived neurotrophic factor (GDNF) receptor alpha-1 (GFRA1) (1:100, ab8026, Abcam, UK), anti-distal-less homeobox 5 (DLX5) (1:100, ab109737, Abcam, UK), anti-cytochrome c oxidase subunit 7A1 (COX7A1) (1:100, 11413-1-AP, Proteintech, USA), anti-interleukin 1 beta (IL-1β) (1:100, NB600-633, NOVUS, USA), anti-collagen I (1:100, A22090, ABclonal, China), anti-collagen II (1:100, ab185430, Abcam, UK) and anti-aggrecan (1:100, 3880-1-AP, Proteintech, USA) antibodies at 4 °C overnight. Nonimmune rabbit or mouse IgG at the same dilution as the primary antibody was used as a negative control. After being washed, the sections were labeled with horseradish peroxidase-conjugated goat anti-rabbit antibody (1:100, a0208, Beyotime, China) or goat anti-mouse antibody (a0216, Beyotime, China) at room temperature for 60 min, after which diaminobenzidine (DAB) (ab64238, Abcam, UK) was used to visualize the chromogen, and hematoxylin was used for counterstaining. After dehydration and clearing with dimethylbenzene, the sections were sealed with neutral gum, and 20 × magnification images were

obtained using a bright-field microscope (CX43, Olympus, Japan) for each section.

Immunofluorescence (IF) staining

Cell slides were fixed with 4% paraformaldehyde (P0099, Beyotime, China) for 15 min, permeabilized with 0.3% Triton X-100 in PBS (PBS-T) for 15 min, and blocked with PBS containing 5% BSA for 1 h. To observe specific expression levels, the samples were incubated with antibodies against collagen I (1:100, A22090, ABclonal, China), collagen II (1:100, ab185430, Abcam, UK), aggrecan (1:100, 13880-1-AP, Proteintech, USA), and p50 (1:200, 13586S, CST, USA) at 4 ℃ overnight. Nonimmune rabbit or mouse IgG at the same dilution as the primary antibody was used as a negative control. After being washed three times with PBS, the cells were incubated with goat anti-rabbit Alexa Fluor 555 (ab150062, Abcam, UK), goat anti-rabbit Alexa Fluor 488 (A-11008, Invitrogen, USA) or goat anti-mouse Alexa Fluor 488 (A-11001, Invitrogen, USA) antibodies at a dilution of 1:500 for 1 h at room temperature. The cells were subsequently washed three times and sealed with an anti-fluorescence quenching agent containing DAPI (P0131, Beyotime, China). The sections were examined and photographed using a fluorescence microscope (CX43, Olympus, Japan).

Differential expression analysis

We calculated differentially expressed genes (DEGs) in each cluster using the limma package in R (v3.50.3). The genes with a P-value < 0.05 and $\log_2$ (fold change) > 0.5 were regarded as signature genes.

Enrichment analysis

Gene Ontology (GO) analysis was conducted on DEGs using the clusterProfiler (v4.2.2) R package, which employs a hypergeometric test with a significance threshold of 0.05 (**Fig. 1d**). Additionally, we assessed the biological processes and signaling pathways of each cluster using gene set variation

analysis (GSVA v1.42.0) and gene set enrichment analysis (GSEA) (clusterProfiler v4.2.2) in R. The gene signatures used for analysis were sourced from Hallmark gene sets, C2 curated gene sets, and C5 ontology gene sets (MSigDB database v7.5.1). Furthermore, a set of matrisome-related genes identified in the IVD was obtained from MatrisomeDB [7].

Matrisome-associated gene interaction network

The core genes of the matrisome in each cluster were defined as the intersections of the upregulated DEGs in each cluster with MatrisomeDB [$\log_2$ (fold change) > 0.5 for C1-C3 and > 1.8 for C4]. The interactions among collagens, proteoglycans, extracellular matrix (ECM) glycoproteins, ECM-affiliated proteins, ECM regulators and secreted factors were predicted using the STRING database [8]. The gene interaction network was visualized using Cytoscape (v.3.9.1) [9].

Single-cell dataset integration analysis

We obtained single-cell RNA-seq (scRNA-seq) datasets from gene expression omnibus databases (GSE160756 [10] and GSE165722 [11]) and performed integration analysis using Harmony in Seurat with the RunHarmony function [12,13]. The identification of the NPC subclusters was based on our previous study [10], and the anchors were computed using the FindIntegrationAnchors function **(Fig. 3a)**. Subclusters of blood cells were identified on the basis of immune cell markers [16] (**Fig. 3b; Additional file 1: Fig. S3a, b**). The expression of marker genes of four subtypes was visualized using dot plots (**Additional file 1: Fig. S3d**).

BayesPrism and CIBERSORT deconvolution to reveal the cell subpopulations of each subtype

To predict the cellular composition of each subtype, we applied BayesPrism deconvolution, which uses bulk RNA-seq data along with a reference single-cell expression dataset to impute gene expression [14]. Raw counts from bulk RNA-seq data were used as input, with the integrated single-cell dataset

serving as the reference. The cellular composition was visualized using a box plot (**Fig. 3c**). Furthermore, to computationally enumerate immune cell types from bulk RNA-seq samples, we used the CIBERSORT algorithm with the leukocyte signature matrix (LM22) gene signature to estimate their relative fractions [15] (**Additional file 1: Fig. S3c**).

Scissor analysis to identify subtype-associated scRNA datasets

Scissor [16] was employed to identify the scRNA datasets associated with each subtype. The original four-class molecular subtype was restructured into a set of binary classification problems, each corresponding to a different subtype against all others. The Scissor pipeline analyzed scRNA-seq data with bulk RNA-seq data grouped into each subtype and other subtypes. The cells were categorized as Scissor-positive (Scissor^{+}) or Scissor-negative (Scissor^{-}) on the basis of their association with each subtype, with the respective cell subpopulations and proportions visualized in **Fig. 3d, e** and **Additional file 1: Fig. S3e**. DEGs between Scissor^{+} cells and all other cells were identified in Seurat (v4.2.0) using the function FindMarkers and visualized using volcano plots (**Additional file 1: Fig. S3e**). GO analysis was conducted on DEGs using the clusterProfiler (v4.2.2) R package, which employs a hypergeometric test with a significance threshold of 0.05.

Intercellular interaction analysis

To reveal the intercellular communication networks of C4 subtype discs, CellChat (v1.5.0) was used for potential ligand – receptor analysis on C4-associated Scissor^{+} cells. Interaction pairs with $P < 0.05$ were considered significant and were retained. The interactions between macrophages and other clusters were further analyzed.

Gene classifier for molecular classification

Screening for significant predictors of classification was conducted using the random forest (RF)

model. We divided the NP samples into a training set and a testing set (7:3). The mean decrease in the Gini coefficient and accuracy from the RF model was used to assess the contribution of each independent variable to classification performance (**Additional file 1: Fig. S1e, f**). Compared with traditional regression methods, the RF method offers robust resistance to interference and reduces sensitivity to outliers, making it particularly effective for handling multilevel categorized variables [17]. Through a tenfold cross-validation experiment, we identified the top 248 mean decrease accuracy variables with the lowest error, which were ultimately included in the prediction model (**Additional file 1: Fig. S1d**). R multiROC (v1.1.1) was used to construct the receiver operating characteristic (ROC) and precision–recall (PR) curves (**Additional file 1: Fig. S3g**). Furthermore, we collected the external data GSE15227 [18], GSE23130 [19], and GSE70362 [20], and our model was able to identify individual clusters in external datasets.

References

1. Chen Y, Chen Y, Shi C, Huang Z, Zhang Y, Li S, et al. SOAPnuke: a MapReduce acceleration-supported software for integrated quality control and preprocessing of high-throughput sequencing data. Gigascience. 2018;7(1):1–6.
2. Kim D, Paggi JM, Park C, Bennett C, Salzberg SL. Graph-based genome alignment and genotyping with HISAT2 and HISAT-genotype. Nat Biotechnol. 2019;37(8):907–15.
3. Liao Y, Smyth GK, Shi W. featureCounts: an efficient general purpose program for assigning sequence reads to genomic features. Bioinformatics. 2014;30(7):923–30.
4. Love MI, Huber W, Anders S. Moderated estimation of fold change and dispersion for RNA-seq data with DESeq2. Genome Biol. 2014;15(12):550.
5. Ren G, Juhl M, Peng Q, Fink T, Porsborg SR. Selection and validation of reference genes for qPCR analysis of differentiation and maturation of THP-1 cells into M1 macrophage-like cells. Immunol Cell Biol. 2022;100(10):822–9.
6. Li XC, Luo SJ, Fan W, Zhou TL, Tan DQ, Tan RX, et al. Macrophage polarization regulates intervertebral

disc degeneration by modulating cell proliferation, inflammation mediator secretion, and extracellular matrix metabolism. Front Immunol. 2022;13:922173.

7. Shao X, Gomez CD, Kapoor N, Considine JM, Grams C, Gao YT, et al. MatrisomeDB 2.0: 2023 updates to the ECM-protein knowledge database. Nucleic Acids Res. 2023;51(D1):D1519–30.
8. Szklarczyk D, Kirsch R, Koutrouli M, Nastou K, Mehryary F, Hachilif R, et al. The STRING database in 2023: protein-protein association networks and functional enrichment analyses for any sequenced genome of interest. Nucleic Acids Res. 2023;51(D1):D638–46.
9. Shannon P, Markiel A, Ozier O, Baliga NS, Wang JT, Ramage D, et al. Cytoscape: a software environment for integrated models of biomolecular interaction networks. Genome Res. 2003;13(11):2498–504.
10. Gan Y, He J, Zhu J, Xu Z, Wang Z, Yan J, et al. Spatially defined single-cell transcriptional profiling characterizes diverse chondrocyte subtypes and nucleus pulposus progenitors in human intervertebral discs. Bone Res. 2021;9(1):37.
11. Tu J, Li W, Yang S, Yang P, Yan Q, Wang S, et al. Single-cell transcriptome profiling reveals multicellular ecosystem of nucleus pulposus during degeneration progression. Adv Sci (Weinh). 2022;9(3):e2103631.
12. Butler A, Hoffman P, Smibert P, Papalexi E, Satija R. Integrating single-cell transcriptomic data across different conditions, technologies, and species. Nature biotechnology. 2018;36(5):411–20.
13. Korsunsky I, Millard N, Fan J, Slowikowski K, Zhang F, Wei K, et al. Fast, sensitive and accurate integration of single-cell data with Harmony. Nat Methods. 2019;16(12):1289–96.
14. Chu T, Wang Z, Pe'er D, Danko CG. Cell type and gene expression deconvolution with BayesPrism enables Bayesian integrative analysis across bulk and single-cell RNA sequencing in oncology. Nat Cancer. 2022;3(4):505–17.
15. Newman AM, Liu CL, Green MR, Gentles AJ, Feng W, Xu Y, et al. Robust enumeration of cell subsets from tissue expression profiles. Nat Methods. 2015;12(5):453–7.
16. Sun D, Guan X, Moran AE, Wu L-Y, Qian DZ, Schedin P, et al. Identifying phenotype-associated subpopulations by integrating bulk and single-cell sequencing data. Nat Biotechnol. 2022;40(4):527–38.
17. Rigatti SJ. Random forest. J Insur Med. 2017;47(1):31–9.
18. Gruber HE, Ingram JA, Hoelscher GL, Zinchenko N, Hanley EN, Sun Y. Asporin, a susceptibility gene in osteoarthritis, is expressed at higher levels in the more degenerate human intervertebral disc. Arthritis Res Ther. 2009;11(2):R47.
19. Gruber HE, Hoelscher GL, Ingram JA, Hanley EN. Genome-wide analysis of pain-, nerve- and neurotrophin-related gene expression in the degenerating human annulus. Mol Pain. 2012;8:63.

20. Kazezian Z, Gawri R, Haglund L, Ouellet J, Mwale F, Tarrant F, et al. Gene expression profiling identifies interferon signalling molecules and IGFBP3 in human degenerative annulus fibrosus. Sci Rep. 2015;5:15662.

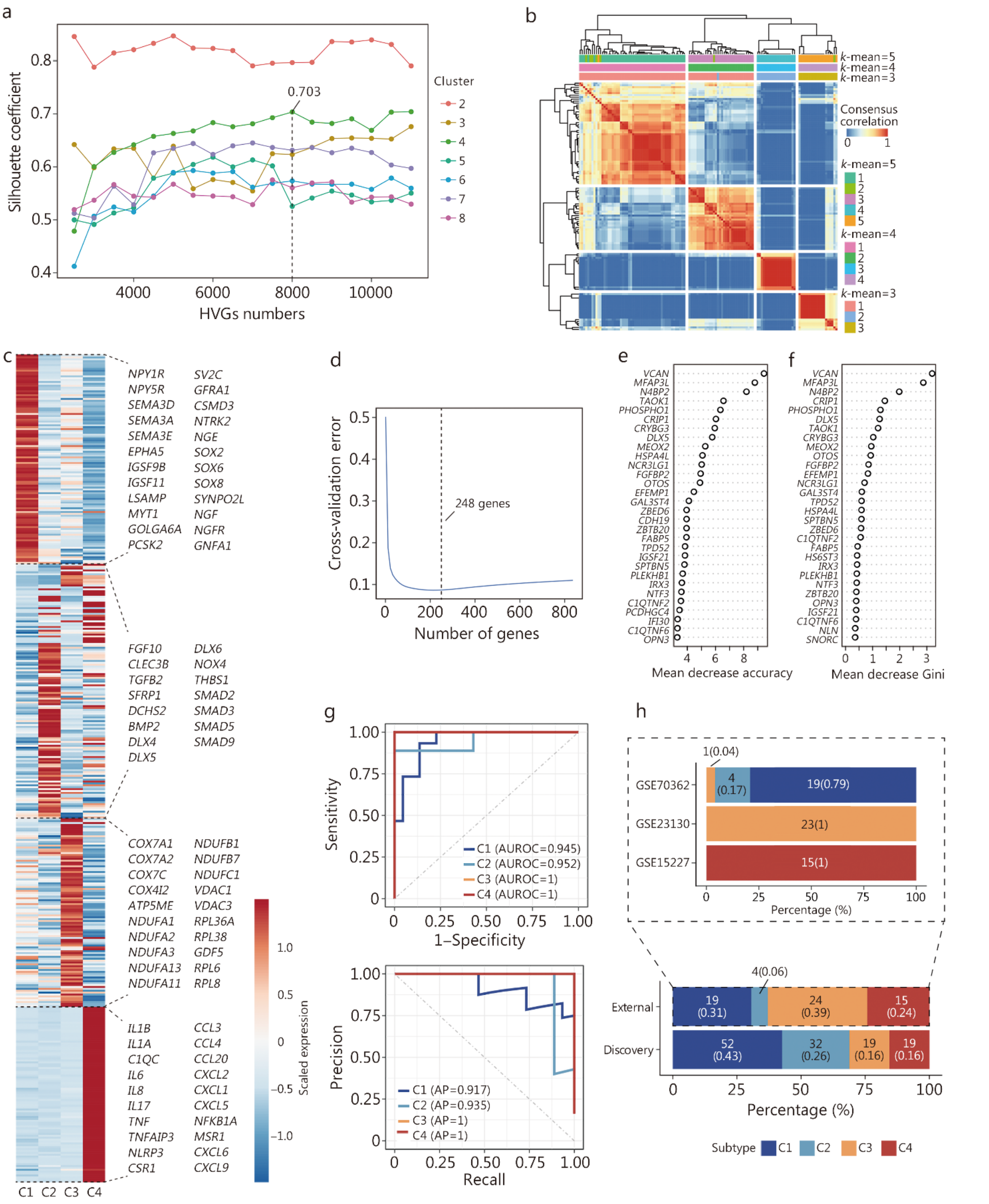

Fig. S1 Molecular classification by unsupervised clustering and external microarray validation. **a** Line chart showing the silhouette coefficient of 2 – 8 clusters on the basis of the top *n* (*n* ranging from 2500

to 11,000, with an interval of 500) HVGs. **b** Heatmap showing 3-5 clusters based on the top 8000 HVGs. **c** Heatmap showing subtype-specific signature genes. RF modeling a gene classifier composed of 248 DEGs (**d**) and the top 30 genes ranked by the mean decrease in accuracy (**e**) and Gini coefficient (**f**). **g** ROC curves, AUROCs, PR curves and APs derived from the discovery cohort. **h** LDD subtype compositions of three external individual microarray datasets and the discovery datasets. C1 cluster 1, C2 cluster 2, C3 cluster 3, C4 cluster 4, DEGs differentially expressed genes, LDD lumbar disc degeneration, HVGs highly variable genes, RF random forest, ROC receiver operating characteristic, AUROC area under the ROC curve, PR precision-recall, AP area under the PR curve

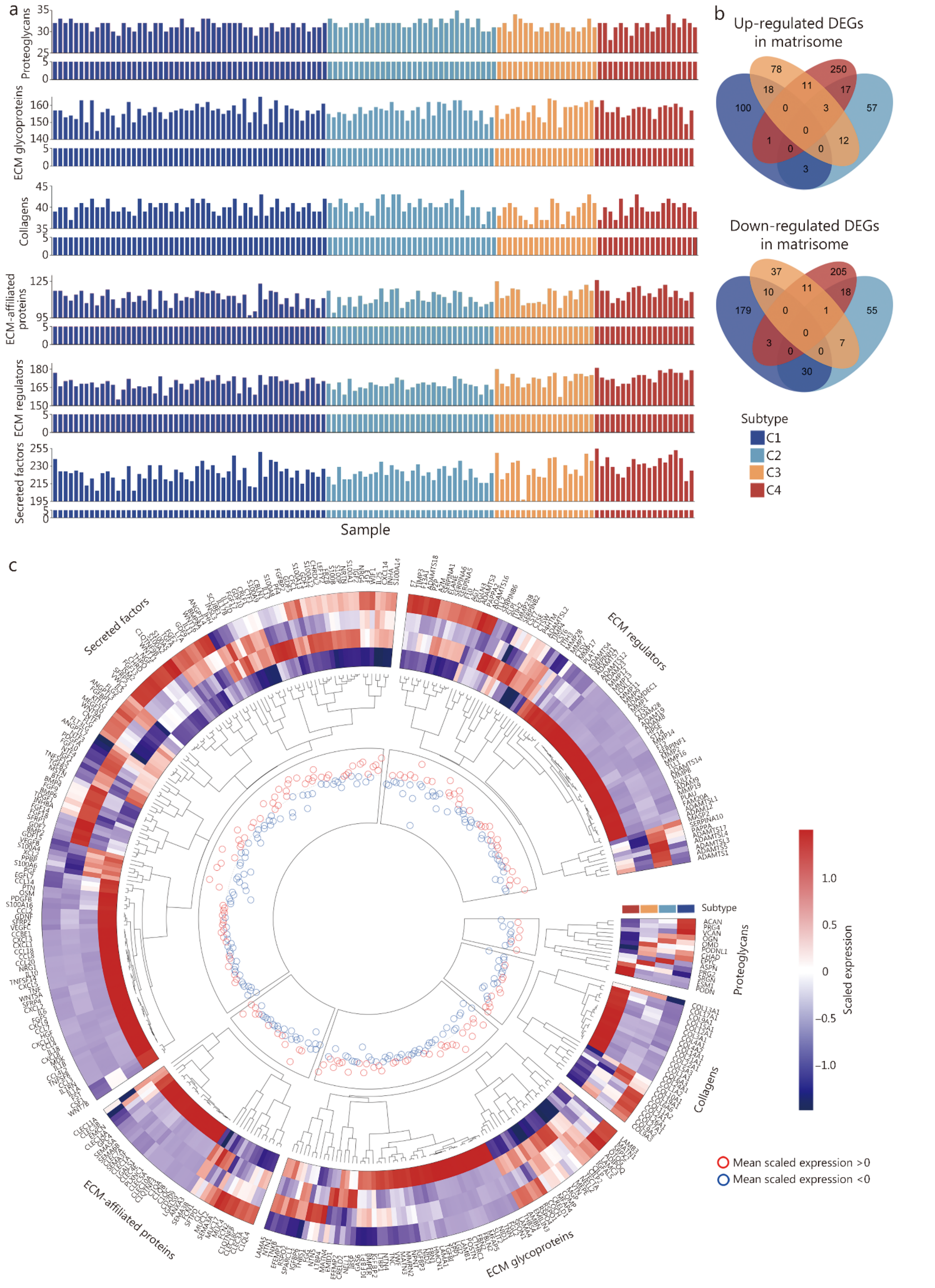

Fig. S2 Attributes of matrisome-associated genes per sample in each subtype. **a** Bar charts showing the composition of the matrisome-associated genes per sample. The height of the bars indicates the

number of genes in each matrisome category expressed per sample. **b** Venn diagram showing the number of DEMGs in each subtype. **c** Heatmap showing the gene expression of six matrisome modules per sample in each subtype. C1 cluster 1, C2 cluster 2, C3 cluster 3, C4 cluster 4, ECM extracellular matrix, DEMGs differentially expressed matrisome genes

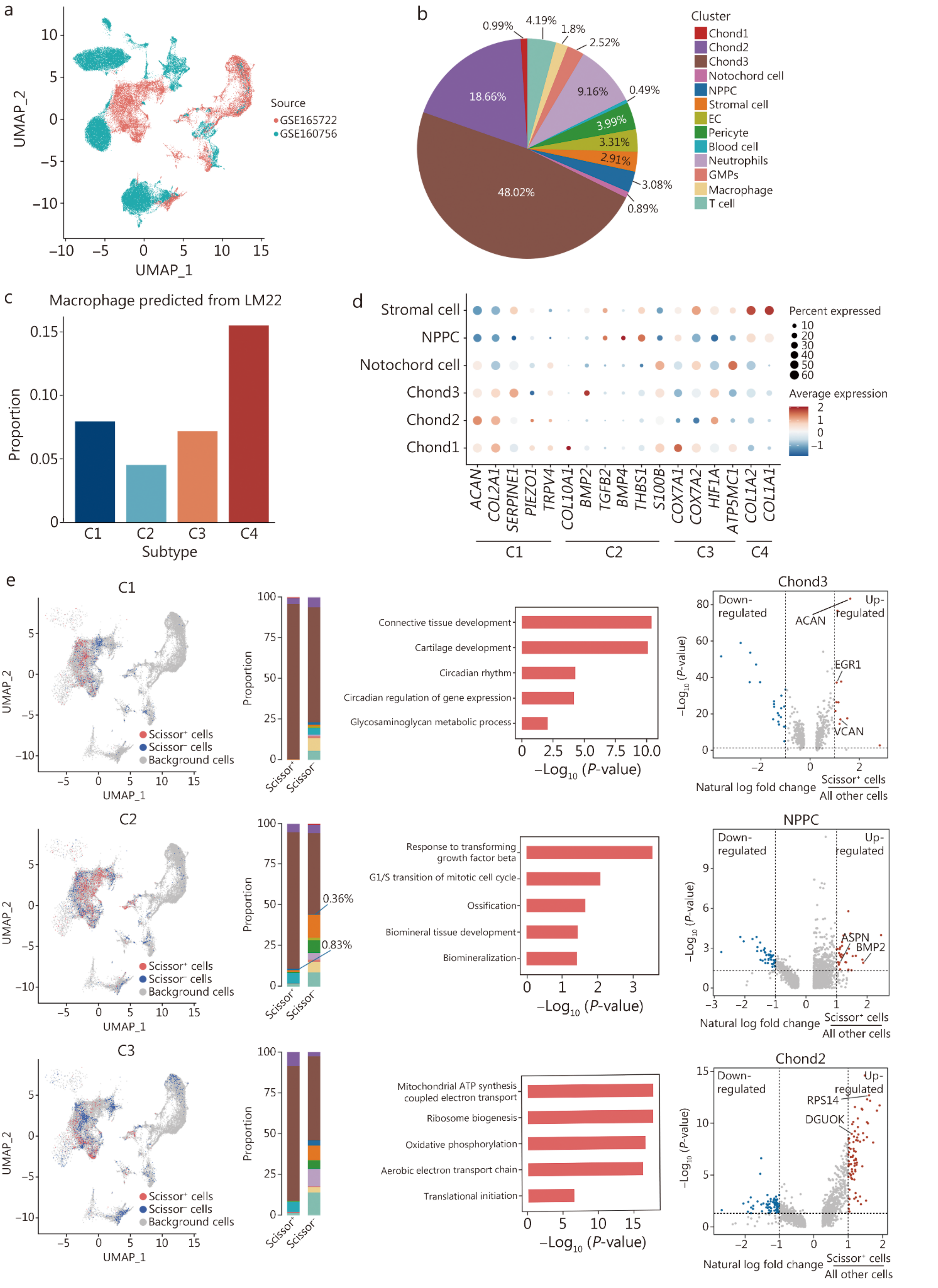

Fig. S3 Subtype-specific cell subpopulations and functional phenotypes. **a** UMAP visualizing integrated scRNA-seq data (GSE160756 and GSE165722). **b** Pie chart showing the proportions of cell

subpopulations. **c** Macrophage proportion in each subtype determined by the CIBERSORT algorithm with the leukocyte signature matrix (LM22) gene signature. **d** Representative DEMGs per subtype mapping the cell subpopulations. **e** Scissor-selected cells of the C1, C2 and C3 subtypes and their associated functional phenotypes. The bar charts show the cell subpopulation composition per subtype. The enrichment bar plots indicate the functional phenotypes of the dominant cell subpopulation per subtype. The volcano plots indicate DEMGs in Scissor$^+$ cells versus all other cells. ATP adenosine triphosphate, C1 cluster 1, C2 cluster 2, C3 cluster 3, C4 cluster 4, CIBERSORT cell-type identification by estimating relative subsets Of RNA transcripts, NPPC nucleus pulposus progenitor cell, EC endothelial cells, DEMGs differentially expressed matrisome genes, GMPs granulocyte-monocyte progenitors, UMAP uniform manifold approximation and projection

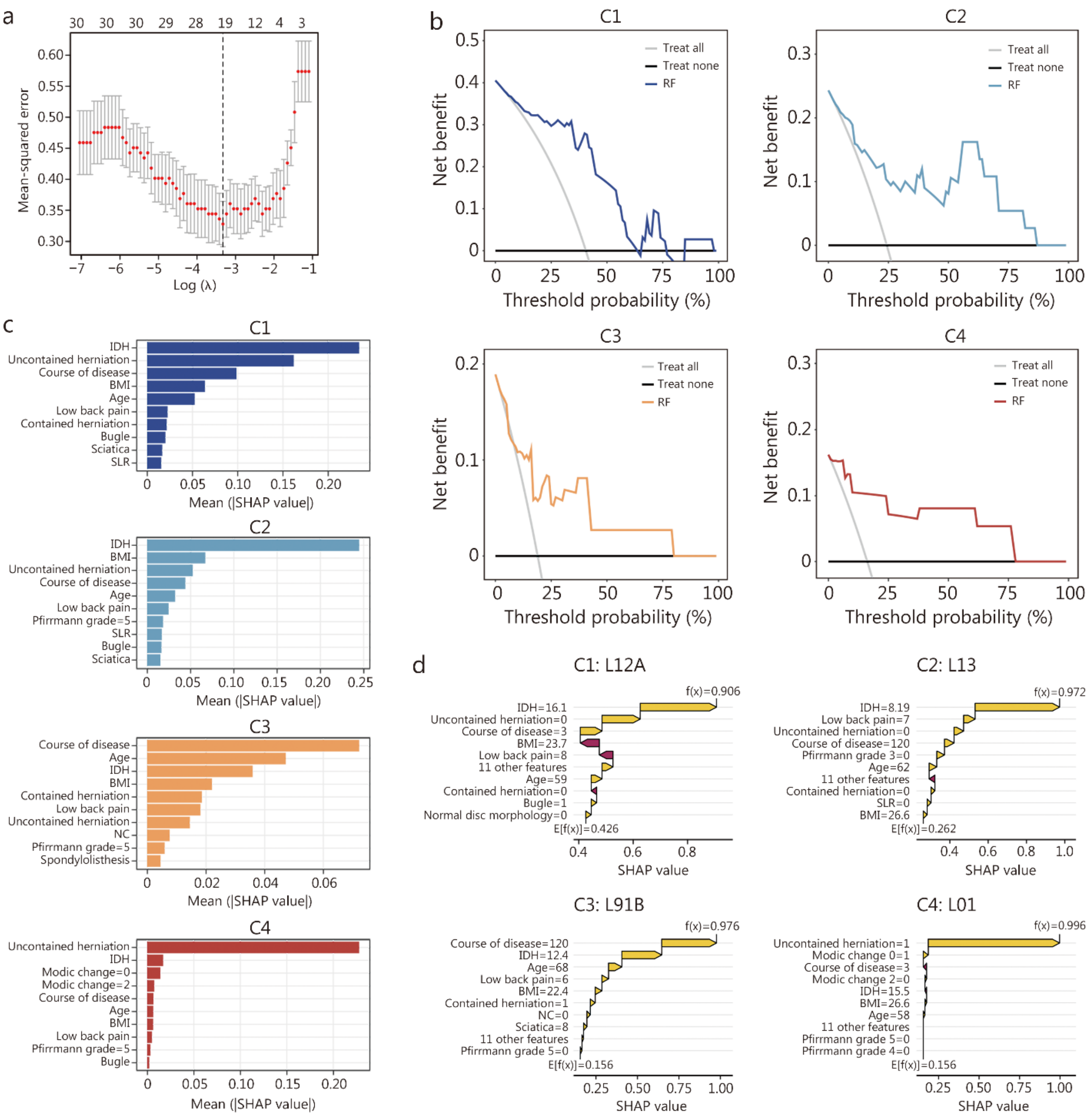

Fig. S4 Feature selection for the machine learning model and clinical usability evaluation and SHAP explanation of the RF model. **a** Log (Lambda) values of the 23 features in the LASSO regression model. A coefficient profile plot was produced against the log (lambda) sequence. **b** Decision curve analysis of the RF model established for each subtype. The abscissa shows the threshold probability, whereas the ordinate shows the net benefit. The gray line represents the assumption of treating all, and the black line represents the assumption of treating none. **c** Feature importance ranking by the mean absolute SHAP value. The matrix diagram describes the importance of each feature for each subtype in the development of the RF model. **d** SHAP model interpretation for representative patients of each subtype. The SHAP value represents the predictive features of four individual patients and the contribution of each feature per subtype. f(x) is the probability forecast value, whereas E[f(x)] is the previous

probability provided to the model. C1 cluster 1, C2 cluster 2, C3 cluster 3, C4 cluster 4, SHAP Shapley additive explanation, LASSO least absolute shrinkage and selection operator, RF random forest, ROC receiver operating characteristic, AUROC area under the ROC curve, IDH intervertebral disc height, BMI body mass index, NC Neurogenic claudication, SLR straight-leg-raising

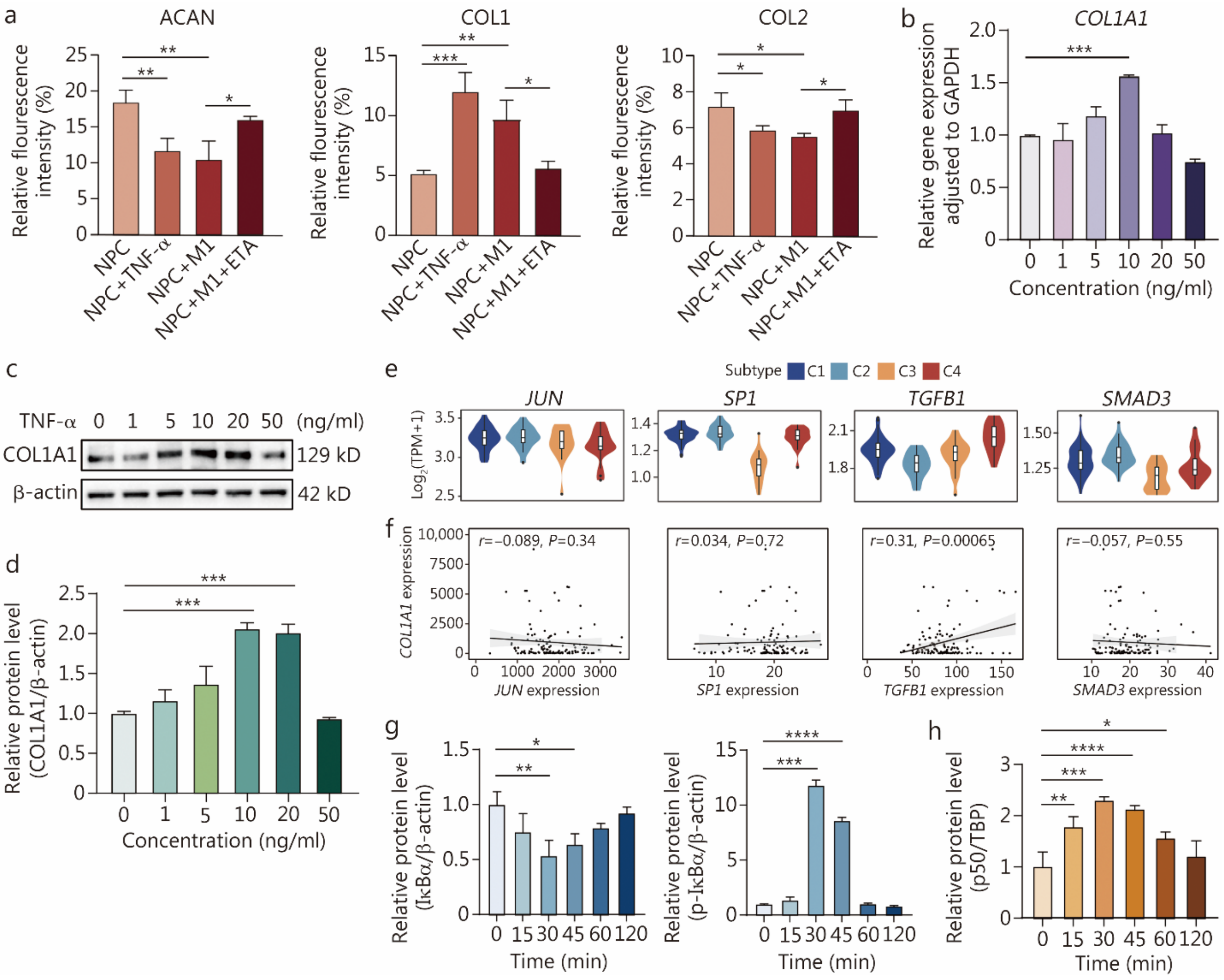

Fig. S5 TNF-α influences COL1A1 expression in INPC in vitro. **a** Bar chart showing the relative fluorescence intensity of ACAN, collagen I (COL1), and collagen II (COL2) in the transwell assay. **b** Bar chart showing the dose-related *COL1A1* expression in INPC treated with TNF-α for 24 h. Immunoblot (**c**) and densitometry plots (**d**) (n = 3) showing the dose-related protein expression of COL1A1 in INPC treated with TNF-α for 24 h. **e** Violin plots showing the expression of *JUN*, *SP1*, *TGFB1*, and *SMAD3* among the four subtypes. **f** Pearson correlation analysis of *COL1A1* with *JUN*, *SP1*, *TGFB1*, and *SMAD3*. Densitometry plots showing the time-dependent expression of IκBα and p-IkBa (**g**) in the cytosolic extracts and NF-κB1 (p50) (**h**) in the nuclear extracts of INPC treated with TNF-α (10 ng/ml) for 24 h. $^{*}P < 0.05$, $^{**}P < 0.01$, $^{***}P < 0.001$, $^{****}P < 0.0001$. NPC nucleus pulposus cell, INPC immortalized NP cell, ACAN aggrecan, TNF-α tumor necrosis factor alpha, TPM transcripts per million, COL1A1 collagen type I alpha 1 chain, *JUN* Jun proto-oncogene, AP1 transcription factor subunit, *SP1* Sp1 transcription factor, *TGFB1* transforming growth factor beta 1, *SMAD3* Smad family member 3, TBP TATA binding protein
